# Kinetic Steering of Amyloid Formation and Polymorphism by Canagliflozin, a Type-2 Diabetes Drug

**DOI:** 10.1021/jacs.4c16743

**Published:** 2025-02-21

**Authors:** Alexander I. P. Taylor, Yong Xu, Martin Wilkinson, Pijush Chakraborty, Alice Brinkworth, Leon F. Willis, Anastasia Zhuravleva, Neil A. Ranson, Richard Foster, Sheena E. Radford

**Affiliations:** † Astbury Centre for Structural Molecular Biology, School of Molecular and Cellular Biology, Faculty of Biological Sciences, University of Leeds, LS2 9JT Leeds, U.K.; ‡ Astbury Centre for Structural Molecular Biology, School of Chemistry, Faculty of Engineering and Physical Sciences, University of Leeds, LS2 9JT Leeds, U.K.

## Abstract

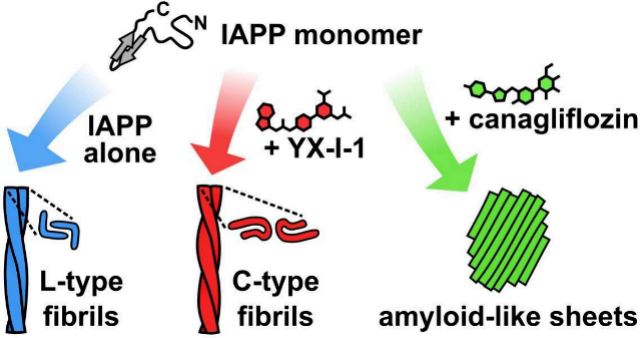

Amyloid formation is involved in widespread health conditions such as Alzheimer’s disease, Parkinson’s disease, and type-2 diabetes. Amyloid fibrils have a similar cross-β architecture, but fibrils formed by a single protein sequence can have diverse structures, varying with time, self-assembly conditions, and sequence modifications. Fibril structure has been proposed to be diagnostic of disease, but why different structures result under different conditions, especially in vitro, remains elusive. We previously identified a small molecule, YX-I-1, which inhibits in vitro amyloid formation by islet amyloid polypeptide (IAPP), a peptide hormone whose amyloid formation is involved in type-2 diabetes. Here, using YX-I-1 as a lead, we identified regulator-approved drugs with similar structures by chemical similarity analysis and substructure searches and monitored the effect of 24 of these potential ligands on IAPP amyloid assembly in vitro. We show that one such compound, canagliflozin (Invokana), a type-2 diabetes drug already in clinical use, can strongly delay the kinetics of IAPP amyloid formation, an activity independent of its intended mode of action [sodium-glucose linked transporter 2 (SGLT2) inhibitor] that may have important therapeutic implications. Combining analysis of amyloid self-assembly kinetics, biophysical characterization of monomer and fibril binding, and cryo-EM of the assembly products, we show that YX-I-1 and canagliflozin target IAPP early in aggregation, remodeling the energy landscape of primary nucleation and profoundly altering the resulting fibril structures. Early binding events thus imprint long-lasting effects on the amyloid structures that form.

## Introduction

Amyloid formation is involved in some of the most societally damaging and rapidly growing causes of morbidity and death worldwide, including Alzheimer’s disease, Parkinson’s disease, and type-2 diabetes.^1,2^ Over 40 different proteins, with diverse native structures or in many cases intrinsic disorder, form amyloid associated with human disease,^3^ yet all amyloid fibrils share a common cross-β architecture consisting of a continuous intermolecular β-sheet supported by networks of zipper and ladder-like interactions.^4^ Islet amyloid polypeptide (IAPP, or amylin) is a 37-residue peptide hormone that is cosecreted with insulin and one of the most amyloid-prone sequences known.^5^ Overproduction and aggregation of IAPP leads to amyloid deposition in the islets of Langerhans, a pathological hallmark of type-2 diabetes.^6−9^ Although the precise link between IAPP aggregation and disease remains uncertain, IAPP aggregates have been shown to be toxic in cell-based assays, and genetic and animal studies implicate islet amyloid in the progressive loss of pancreatic β-cell function.^5^ Therefore, IAPP has been suggested to play a role in the onset of type-2 diabetes,^8,9^ making it a promising target for development of new drugs for this condition.

Recent successes in antibody therapies for Alzheimer’s disease, which target amyloid aggregates of the amyloid-β (Aβ) peptide, have exemplified the therapeutic potential of targeting amyloid.^10^ The cross-β structures of amyloid fibrils, with a characteristic 4.8 Å longitudinal repeat, deep surface grooves, and ladders of repeating side chains,^4^ are also amenable to small molecule binding.^11,12^ Many small molecules are known to recognize amyloid, with some binding generically [famously thioflavin T (ThT)^13^ and Congo red^14^], and others binding more specifically, such as morphology-sensitive dyes^15−17^ and tracers used for positron emission tomography (PET) imaging in the clinic.^18^ Small molecule inhibitors of amyloid formation can also target the monomeric state of the amyloid precursor, or early aggregation intermediates involved in disease, although only one such molecule, tafamidis for transthyretin amyloidosis,^19^ is currently in the clinic. The hunt is on to find similarly effective small molecule modulators of amyloid formation by IAPP and other proteins.^20^

A major challenge in developing modulators of amyloid assembly is the structural diversity of species formed during aggregation and the wide range of fibril structures that can result.^21^ In contrast to globular proteins, whose folded structure corresponds to a (usually) unique energy minimum, amyloid fibrils formed by a single sequence can adopt a wide range of amyloid folds, termed “polymorphs”, with the resulting structure(s) critically dependent on the protein sequence, post-translational modifications, and solution or cellular conditions.^21^ Thus, amyloid formation is under kinetic rather than thermodynamic control, as exemplified by recent cryo-EM studies showing that amyloid fibril structure changes throughout the course of assembly.^22,23^ Amyloid polymorphism raises fundamental questions about the molecular mechanisms of assembly and the culprits of cytotoxicity in amyloid disease. A recent study demonstrating that a single post-translational modification diverts assembly of α-synuclein into a relatively nontoxic, nonspreading amyloid form suggests that small molecules could be used to redirect assembly toward nontoxic products.^24^ Given the known link between amyloid structure and disease phenotype (at least for tauopathies and synucleinopathies),^25^ inhibitors that leave a lasting fingerprint on polymorphism could provide new routes to therapeutic development.

Here, we build on previous work in which we identified YX-I-1, an inhibitor of IAPP amyloid formation.^26^ YX-I-1 was discovered by screening a focused library of ∼1500 small molecules, using native mass spectrometry and ThT assays, for compounds able to bind IAPP and modulate (accelerate or inhibit) its amyloid formation in vitro*.*^26^ Here, we carried out ligand-based virtual screening to identify regulator-approved drugs that are shape-wise and/or substructurally similar to YX-I-1 and tested their ability to inhibit IAPP amyloid assembly in vitro. Our multitiered screen showed that canagliflozin (Invokana), already in use as a type-2 diabetes drug,^27^ strongly inhibits IAPP amyloid formation, while related molecules in the same class have little, or no, effect. The inhibitory activity of canagliflozin is unrelated to its intended mode of action as a sodium-glucose-linked transporter 2 (SGLT2) inhibitor,^27^ revealing an unexpected dual activity that may have therapeutic implications. Exploiting a detailed kinetic, biophysical, and structural investigation of IAPP amyloid assembly in the presence of YX-I-1 or canagliflozin, we show that these small molecules bind monomers and early species in aggregation, altering the energy landscape of nucleation and diverting assembly to a new amyloid product with a different architecture. Our results show that molecules that modulate the kinetics of amyloid formation can have a profound, long-lasting impact on the amyloid structure.

## Results

### Virtual Screening Identifies Regulator-Approved Drugs with Structural Similarity to YX-I-1

**Figure 1:**
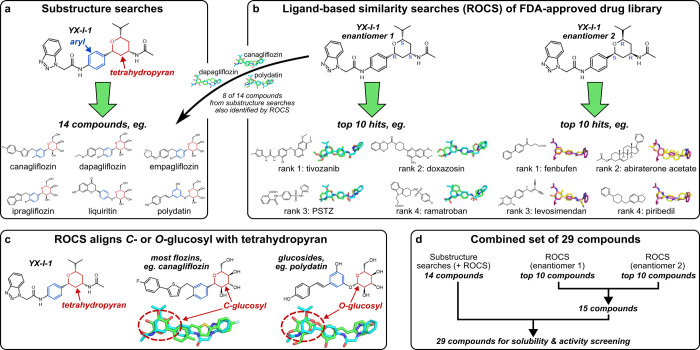
Virtual screening identifies regulator-approved drugs with structural similarity to YX-I-1. (a) Identification of 14 regulator-approved compounds with similarity to YX-I-1 based on substructure searches for the tetrahydropyran (highlighted in red), with example structures below. Notably, in all cases, the tetrahydropyran is *C*- or *O*-linked to an aromatic ring (blue). (b) Ligand-based similarity screening using ROCS (OpenEye Scientific) of the two enantiomers of YX-I-1 against an FDA-approved drug library (SelleckChem, 3008 compounds) with example alignments (cyan, enantiomer 1; purple, enantiomer 2; green/yellow, aligned compound). Note that our previous studies on IAPP amyloid inhibition by YX-I-1 utilized a mixture of both enantiomers.^26^ 8 of the 14 compounds from substructure searches were also identified by the ROCS-based search, but ranked outside the top 10 for either enantiomer. (c) For compounds identified by both methods, the ROCS-based search aligned the tetrahydropyran of YX-I-1 with a *C*- or *O*- glucosyl in the hit compound. (d) All three compound sets were combined to yield 29 compounds for further investigation.

We set out to identify drug-like small molecules that inhibit IAPP amyloid formation, building on our previous success in identifying YX-I-1 as a lead.^26^ Specifically, we sought to identify compounds that have been approved for other uses by medical regulators, possess structural similarity to YX-I-1, and could be repurposed as IAPP amyloid assembly inhibitors. This involved two complementary approaches: (i) substructure searches based on a preliminary structure–activity relationship (SAR) of YX-I-1 against commercially available compound libraries (Figure 1a) and (ii) ligand-based similarity searches using Rapid Overlay of Chemical Structures (ROCS) (OpenEye Scientific) (Figure 1b), which compares the shape and pharmacophoric properties of small molecules irrespective of substructure.^28^ For the substructure searches, we identified regulator-approved compounds that contain a tetrahydropyran ring, as preliminary SAR had suggested that the tetrahydropyran-containing part of YX-I-1 is important, although not necessarily sufficient, for the activity of YX-I-1 (Figure S1). After excluding polyphenols, which have poor drug-like properties, this yielded a set of 14 readily available tetrahydropyran-containing compounds, of which 10 have been FDA-approved, and the remaining 4 have been approved by other regulators (Table S1). In all identified compounds, the tetrahydropyran occurred as part of a glucosyl moiety *C*- or *O*-linked to an aromatic ring (Figure 1a). For the ligand-based similarity searches using ROCS, we screened the FDA-approved drug library (SelleckChem, 3008 compounds) and selected the 10 highest-ranked compounds by similarity (ComboScore) to each of the two enantiomers of YX-I-1, which yielded a set of 15 compounds (Tables S2 and S3) after accounting for overlaps (i.e., compounds that were similar to both enantiomers), and excluding two molecules that were PEGylated or contained a disulfide bond and were thus unsuitable for use in in vitro self-assembly assays (Methods, Table S2). Although the two final sets of virtually screened compounds were distinct, 8 of the 14 compounds from the substructure search were also identified by ROCS, with a rank in the top 10% for either enantiomer. In these cases, ROCS usually aligned the tetrahydropyran ring and adjacent aromatic ring to equivalent moieties in YX-I-1 (Figure 1c), helping to validate the pharmacophoric relevance of the tetrahydropyran-containing compound set. Many of the tetrahydropyran-containing compounds were flozins, a class of drugs that inhibit SGLT2, a transporter involved in glucose reuptake in the kidneys. Beyond the flozins and other tetrahydropyran-containing compounds, the combined set of 29 compounds (Figure 1d) covered a broad chemical space and mostly had excellent drug-like properties (Table S4).

### Canagliflozin and Doxazosin Inhibit IAPP Amyloid Formation

**Figure 2:**
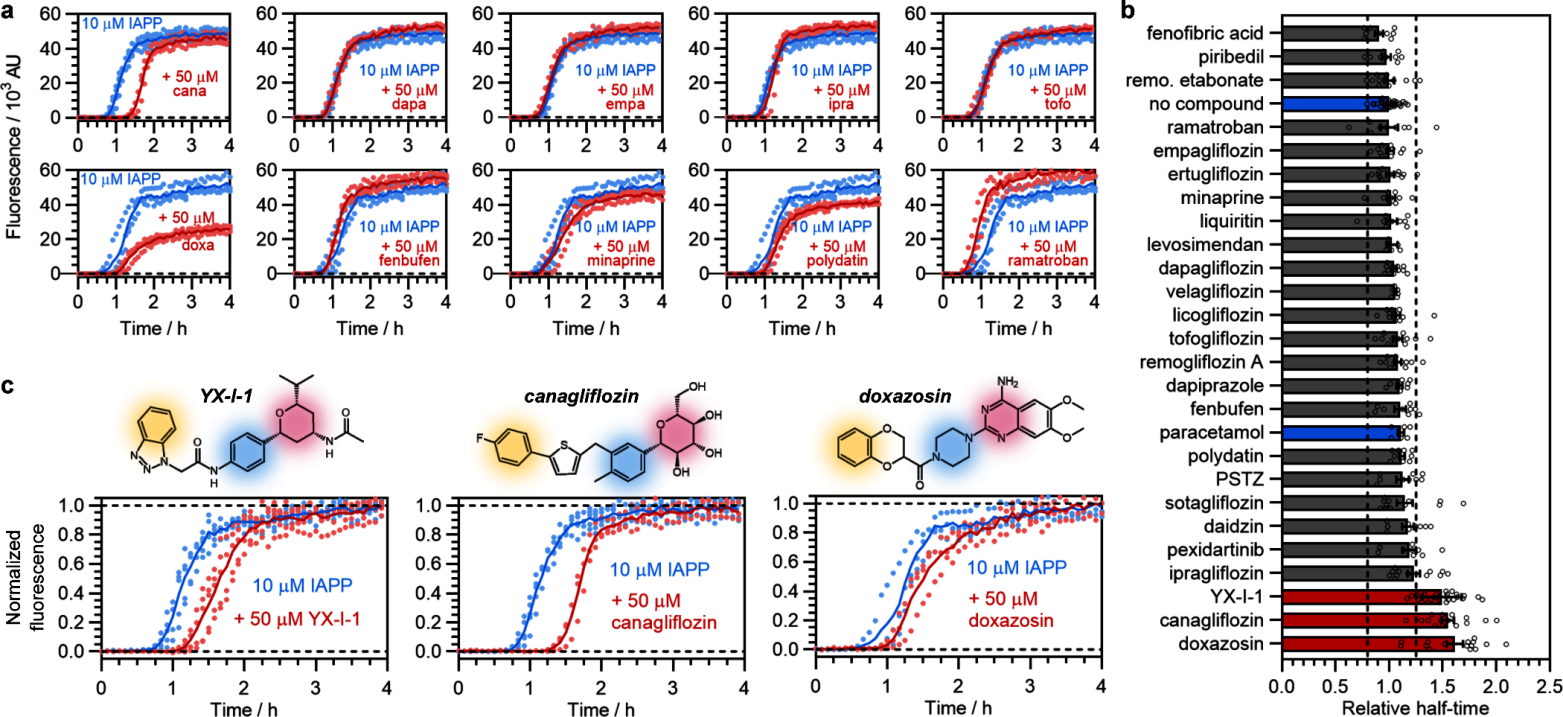
Canagliflozin and doxazosin inhibit IAPP aggregation. (a) Examples of activity screening for inhibition of amyloid formation using ThT fluorescence. Plots are representative examples of the effects of selected compounds with three replicate wells per compound. Reactions were performed using 10 μM IAPP, in 160 mM ammonium acetate, pH 7.4, 1% v/v DMSO (quiescent). Color scheme: blue, IAPP alone; red, plus compound. (b) Summary of the fold-change in the half-time of amyloid formation for all screened compounds. Data for EGCG, which was analyzed separately, are shown in Figure S6. Each dot represents a fluorescence measurement from a single replicate well, and experiments were performed on at least two plates per compound with at least three replicate wells per plate. Error bars show the standard error of the mean across all replicates. Activity was determined by fold-change and Mann–Whitney *U* tests, conducted on the full data set and two subsamples to eliminate potential biases (Methods, Figure S6). Three compounds (YX-I-1, canagliflozin, doxazosin) were deemed active, with *p* < 0.0001 across the full data set. Color scheme: blue, negative controls (no compound, or paracetamol); gray, inactive compounds; red, active compounds. Dashed lines depict fold changes of 0.8× and 1.25× used as thresholds for determining activity. (c) Comparison of the structures and effects of YX-I-1, canagliflozin, and doxazosin on IAPP self-assembly kinetics. Coloring of the compound structures indicates regions that were aligned by ROCS. Plots show the normalized ThT kinetics from the above screen (Methods), with the color scheme: blue, IAPP alone; red, plus compound. The YX-I-1 inhibition kinetics from the preliminary SAR (Figure S1), which were conducted under identical conditions, are included in panels b and c for comparison.

Next, we set out to determine whether any of the 29 compounds from the virtual screening could inhibit IAPP amyloid formation. First, we carried out solubility screening, using absorbance spectroscopy from 235 to 700 nm (to identify light scattering) and flow-induced dispersion analysis (FIDA) (Methods) (Figure S2). These analyses showed that 5 compounds had poor solubility at concentrations of 5–50 μM in the buffer used for the IAPP self-assembly assays, as evidenced by light scattering or formation of demixed particles, so these were excluded from further investigation (Figures S3 and S4, Table S5). The remaining 24 compounds were screened for inhibition of IAPP amyloid formation using ThT assays (10 μM IAPP, 50 μM compound, in 160 mM ammonium acetate adjusted to pH 7.4 with ammonia solution, containing 1% v/v DMSO and 20 μM ThT, at 30 °C in low-binding 96-well microplates) (Methods) (Figure 2). In this buffer, which was chosen to maximize compatibility with potential downstream experiments, IAPP exhibits rapid amyloid fibril assembly via a mechanism dominated by secondary pathways, in agreement with previous work^26,29,30^ and the ThT signal is proportional to the fibril mass (Figure S5 and Table S6). For controls, IAPP was incubated with 1% v/v DMSO only (negative), 50 μM paracetamol (negative), or 50 μM EGCG (positive^31^). All compounds were tested in 2–5 biological repeats, with at least 3 replicate wells per experiment. A high degree of reproducibility was observed in all cases (Methods).

Representative amyloid assembly kinetics of IAPP with and without small molecules are shown in Figure 2a, and the effects of compounds on the half-time of assembly are summarized in Figure 2b (see also Table S7 and Figure S6). Of the 24 screened compounds, 22 had no effect on the half-time, as did the negative control, paracetamol. However, two of the small molecules—doxazosin, an alpha-1 blocker used to treat hypertension and benign prostatic hyperplasia, and canagliflozin, one of the three most widely used flozins for treatment of type-2 diabetes via its action as an SGLT2 inhibitor—caused a pronounced and statistically significant inhibition (fold-change in half-time of 1.61 ± 0.29, *p* < 0.0001 and 1.55 ± 0.22, *p* < 0.0001, respectively), comparable to the inhibition observed for YX-I-1 (fold-change in half-time of 1.49 ± 0.18, *p* < 0.0001) (Table S7 and Figure S6). Canagliflozin and doxazosin are structurally distinct, but both possess a high degree of structural similarity to YX-I-1 enantiomer 1 as assessed by ROCS, with ComboScores of 0.759 (Table S1) and 0.864 (Table S2), respectively. The presence of shared tetrahydropyran and phenyl substructures makes this similarity particularly obvious for canagliflozin (Figures 1c and 2c), but comparison of the ComboScores indicates that doxazosin nonetheless has a higher overall degree of shape and pharmacophoric similarity to YX-I-1, despite the aligned substructures being different (Figure 2c). The small number of hits, despite the compounds’ structural similarity, implies a high degree of specificity (Figure 2c). Consistent with this, under the same experimental conditions, only canagliflozin was able to inhibit amyloid formation by the familial S20G variant of IAPP (involved in earlier and more severe type-2 diabetes^32^), and none of the three compounds inhibited amyloid formation by the Aβ(1–42) peptide (similar in sequence and length to IAPP) (Figure S7).

### Canagliflozin and YX-I-1 Are Potent Inhibitors of Primary Nucleation

**Figure 3:**
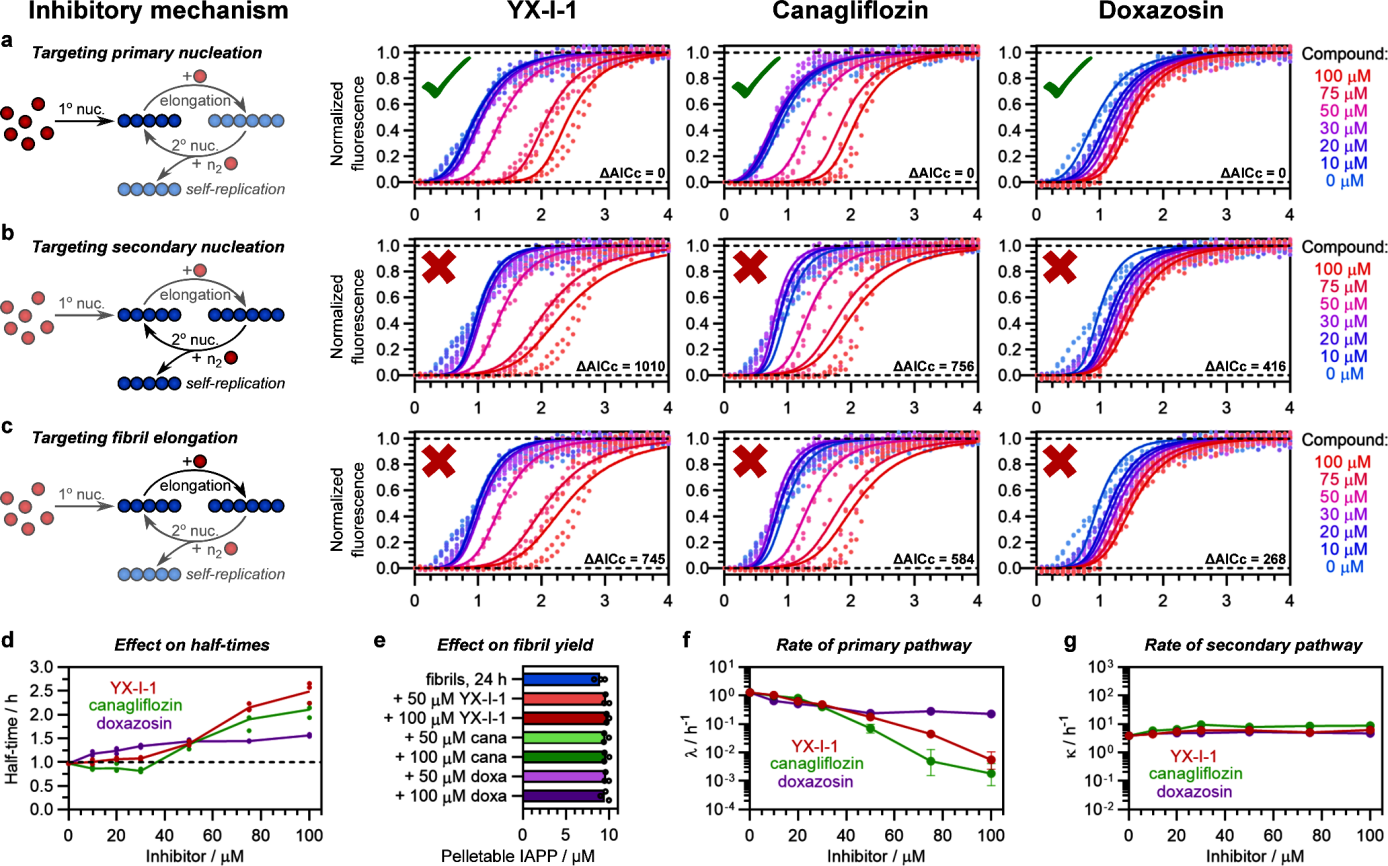
YX-I-1, canagliflozin, and doxazosin are kinetic inhibitors that predominantly target primary nucleation. The dominant mechanism of inhibition of each compound was determined by global fitting of the IAPP self-assembly kinetics from ThT assays with varying concentrations of compounds. Reactions were performed using 10 μM IAPP in 160 mM ammonium acetate (pH 7.4) with 1% v/v DMSO (quiescent). The tested scenarios were: (a) effects on primary nucleation only, (b) effects on secondary nucleation only, and (c) effects on elongation only. In each case, one microscopic process was allowed to vary across compound concentrations, and the rates of all other processes were fitted globally. Fitting strongly favored targeting of primary nucleation over secondary nucleation or elongation (Methods), as quantified by Akaike’s corrected information criterion (AICc; Table S8). The ΔAICc values in the plots are the difference in AICc values relative to the most favored model. The color scheme for the kinetic curves in panels a–c corresponds to the compound concentration, and is shown on the right of each panel. (d) Comparison of the effects of the three compounds on the half-times of IAPP self-assembly. (e) Comparison of the effects of compounds on the fibril yield, as determined by HPLC analysis, confirming that inhibition is of kinetic, rather than thermodynamic, origin. Circles are repeats, and bars represent the mean. (f, g) Direct extraction of the rates of the primary (λ) and secondary (κ) pathways of amyloid fibril assembly, with fitting errors, confirmed that all three compounds mainly target the primary pathway. To allow a comparison of the relative effects on λ and κ, both plots are shown on a logarithmic scale spanning the same number of orders of magnitude. The effects on κ are also shown on an expanded linear scale in Figure S8b. The color scheme for panels d–g corresponds to the small molecule added: red, YX-I-1; green, canagliflozin; purple, doxazosin.

To better understand the mechanisms of IAPP self-assembly inhibition by canagliflozin and doxazosin, and to compare these with the previously reported activity of YX-I-1 (obtained under different assembly conditions to those used here^26^), ThT assays were repeated under the same conditions as above, but with a range of compound concentrations from 0 to 100 μM, and the data were fitted to different models of inhibition (Figure 3a–c, Methods). First, we examined the overall effect on the self-assembly kinetics and the fibril yield. As shown in Figure 3d, YX-I-1 and canagliflozin have similar effects on fibril growth kinetics, with negligible inhibition at concentrations up to 30 μM, but highly dose-dependent inhibition above 30 μM of each small molecule. Note also that a small acceleration in fibril formation is observed in the presence of canagliflozin at low concentrations of the small molecule (half-time is decreased 1.2 times at 30 μM canagliflozin). In contrast, doxazosin is weakly inhibitory at all concentrations, although most of its effect accumulates at concentrations ≤50 μM, with little dose dependence above 50 μM. In addition, doxazosin caused a dose-dependent reduction in the ThT fluorescence intensity end point, which was not observed for the other two compounds (Figures 2a and S8a). To ascertain whether the compounds affect the fibril yield, the contents of the plate wells containing IAPP incubated with 0, 50, or 100 μM compound were collected after 24 h, centrifuged to remove aggregated material, and analyzed by reversed-phase HPLC to quantify the remaining soluble IAPP (Methods). As shown in Figure 3e, none of the compounds had a significant effect on the final yield of pelletable material, despite the effect of doxazosin on the ThT fluorescence intensity end point. Centrifuging the samples at intermediate time points between the lag and early plateau phase and quantifying the remaining soluble material confirmed that all three compounds delay the loss of monomers from solution, consistent with the increase in lag phase observed by ThT fluorescence (Figure S9). Hence the reduction in ThT fluorescence end point observed with doxazosin is an additional effect not directly related to its effect on the half-time, likely due either to competition with ThT for fibril binding sites or a reduced quantum yield of ThT in the fibril-bound state. Thus, all three compounds have a kinetic, rather than thermodynamic, mode of action, altering the rate of fibril formation without affecting the final fibril yield.

Kinetic inhibitors of amyloid formation can target different microscopic processes within the self-assembly pathway, typically primary nucleation, secondary nucleation, and/or elongation.^33^ To determine which of these processes is most affected by canagliflozin and doxazosin, and how these compounds compare with the previously published inhibitor YX-I-1,^26^ the kinetics of amyloid formation in the presence of the three inhibitors were globally fitted to mathematical models reflecting scenarios wherein only primary nucleation (Figure 3a), secondary nucleation (Figure 3b), or elongation (Figure 3c) is affected, with the rates of all unaffected microscopic processes fitted globally across compound concentrations (Methods). For all three compounds, perturbation of primary nucleation was strongly favored over the other two models (Figure 3a–c, Table S8), consistent with the observation that the compounds mainly affect the lag time. This suggests that the compounds’ dominant, although not necessarily exclusive, mode of action is by targeting primary nucleation. Consistent with this conclusion, direct extraction of the macroscopic rate parameters λ and κ, which reflect the collective rate at which monomers are incorporated into fibrils under the influence of primary nucleation and elongation, or secondary nucleation and elongation, respectively,^34,35^ showed that all three compounds reduce λ, but have much less effect on κ under these conditions (Figure 3f–g, Methods). The reduction in λ was particularly strong for canagliflozin and YX-I-1, whereas doxazosin had a weaker effect due to limited dose-dependence above the original screening concentration of 50 μM. The effects on κ are much smaller than the effects on λ, but closer examination (Figure S8b) showed that canagliflozin causes an approximately 2-fold increase in κ at concentrations above 30 μM, with a dose-dependent effect on κ at lower concentrations. This effect, which was not observed for YX-I-1 or doxazosin, explains the weak acceleration of IAPP fibril assembly observed at subinhibitory concentrations (Figure 3d). The ability of canagliflozin to simultaneously inhibit the primary pathway and weakly enhance the secondary pathway suggests that it has a complex mechanism of action, either binding to multiple distinct species in the self-assembly pathway, or causing a change in fibril polymorphism that has a knock-on effect on the rate of the secondary pathways.

### YX-I-1, Canagliflozin, and Doxazosin Bind IAPP Monomers

**Figure 4:**
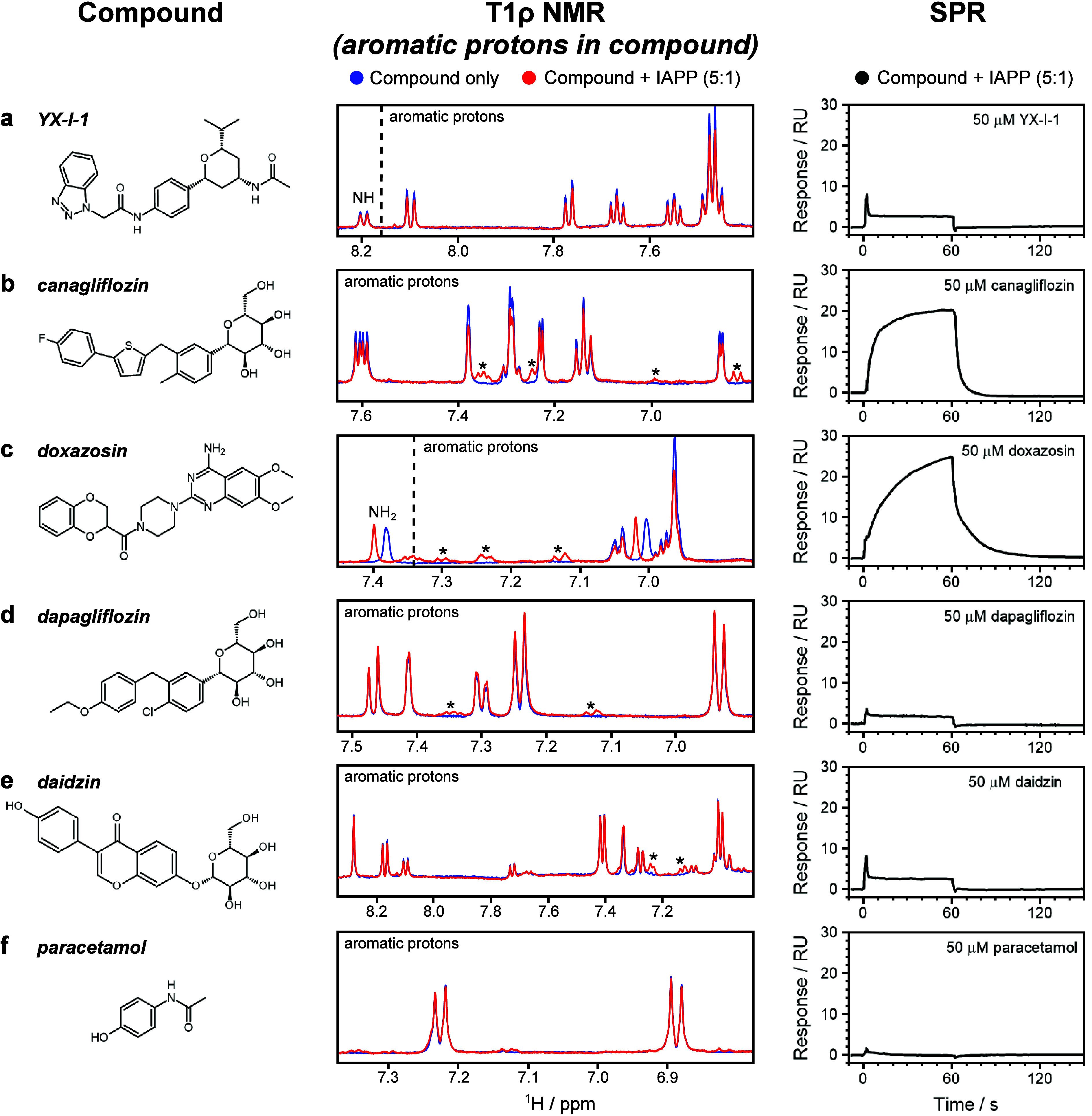
Identification of small molecule binding to IAPP monomers by T1ρ NMR and SPR experiments. (a–f) Binding assays of different compounds (left) to IAPP monomers by T1ρ NMR (center) and SPR (right). Each T1ρ NMR spectrum is representative of a 100 μM small molecule in the absence or presence of 20 μM IAPP, in 25 mM sodium phosphate buffer (pH 6.8) with 1% v/v DMSO. For clarity, only the aromatic region is shown, although the same effect was observed for all protons in each individual experiment. In T1ρ NMR, binding is observed as a reduction of the ^1^H peak intensity (and in some cases chemical shift perturbations) of compound in the presence of IAPP (red), compared to compound alone (blue). The * indicates additional signals that originate from IAPP itself when IAPP is present (red) rather than the small molecule. Each SPR trace is the mean of referenced, blank-subtracted data from three highly concordant repeats, using 10 μM IAPP and 50 μM small molecules, in 160 mM ammonium acetate (pH 7.4) with 1% v/v DMSO. Note that the conditions used here for the NMR experiments differed from those for SPR and were chosen to ensure that the IAPP remained monomeric throughout NMR data acquisition. Controls showed that YX-I-1, canagliflozin, and doxasozin inhibit amyloid assembly in both buffers employed (Figure S10).

To determine whether canagliflozin, and other molecules from the screen, interact with IAPP monomers, surface plasmon resonance (SPR) and T1ρ NMR experiments^36^ were performed (Methods). While the SPR experiments show the density of compound that binds to IAPP immobilized on a sensor chip, T1ρ NMR experiments report on changes in the relaxation rates of the compound due to interactions with IAPP in solution. The results are summarized in Figure 4. Interestingly, all three inhibitors (YX-I-1, canagliflozin, and doxazosin) demonstrated a clear reduction (15–20%) in the intensity of both aromatic and aliphatic protons in T1ρ NMR experiments. This reduction suggests that these compounds bind to the IAPP monomer, enhancing proton relaxation. In contrast, the three inactive controls (dapagliflozin, daidzin, and paracetamol) showed minimal attenuation (<5%), indicating a lack of binding. This suggests that there is a relationship between monomer binding and inhibition of amyloid formation. The same trend was observed for the SPR, with the exception of YX-I-1, which has previously been shown to bind more weakly in SPR compared to solution-based experiments, likely due to the biotinylation of Lys1.^26^

To gain more information on the affinity with which canagliflozin binds IAPP, the SPR experiments were repeated at canagliflozin concentrations ranging from 10 to 150 μM (higher concentrations could not be used owing to the solubility limit of canagliflozin in the buffer used). A dose-dependent SPR response was observed, but the plateau intensity remained linearly proportional to the canagliflozin concentration over the entire concentration range (Figure S11a), suggesting that the interaction is weak under the conditions used in our kinetic assays (*K*_d_ ≫ 150 μM). Consistent with this, global fitting of the SPR traces with a one-to-one binding model failed to converge on a *K*_d_ value (Figure S11b).

### Molecular Basis of the Interaction between Canagliflozin and IAPP Monomers

**Figure 5:**
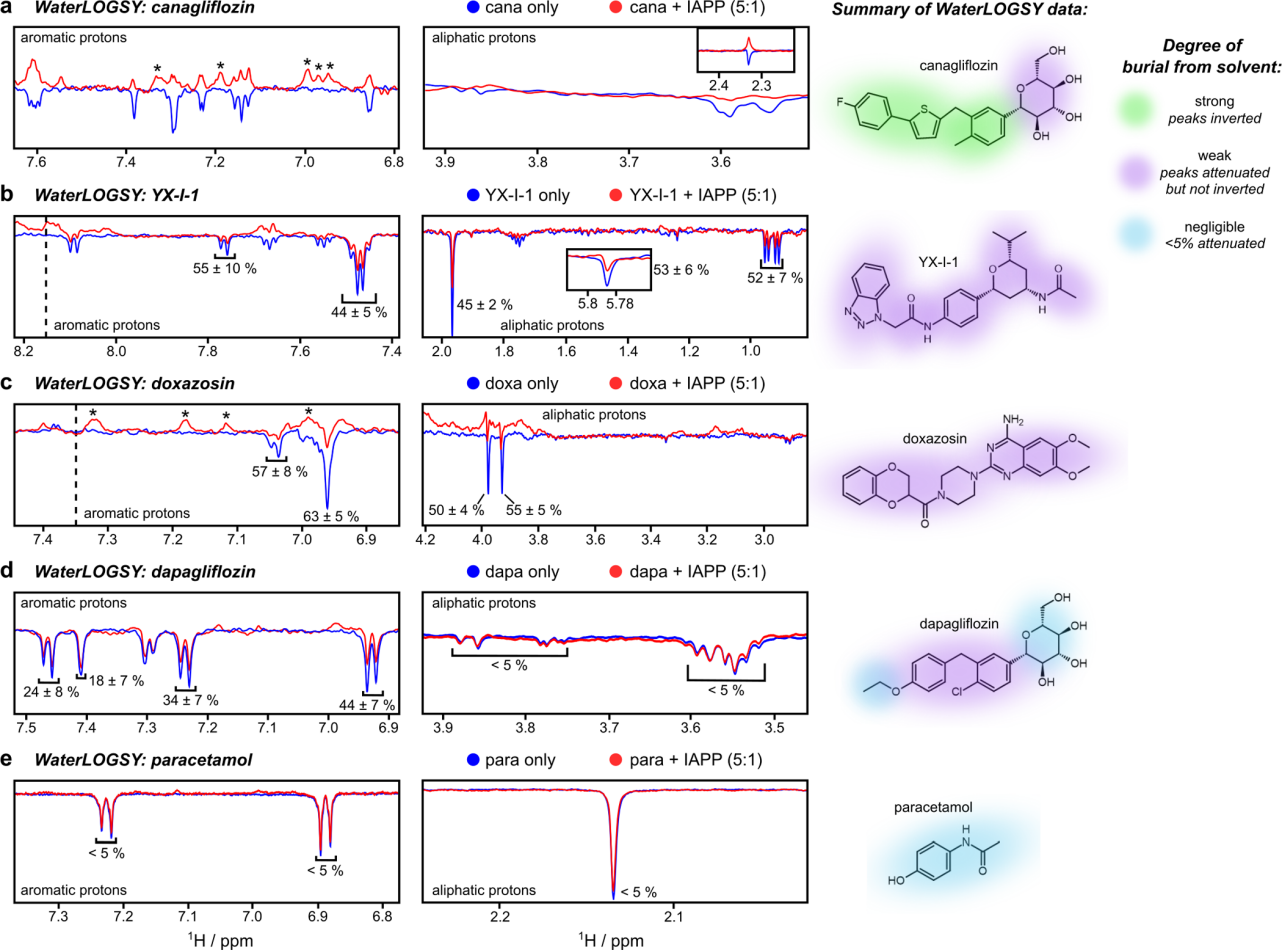
Characterization of small molecule binding to the IAPP monomer using WaterLOGSY NMR. (a–e) Example WaterLOGSY NMR spectra for canagliflozin, YX-I-1, doxazosin, dapagliflozin, and paracetamol (negative control) in the presence or absence of IAPP. Each experiment contained 20 μM IAPP and 100 μM small molecule, in 25 mM sodium phosphate (pH 6.8), with 1% v/v DMSO. The schematics on the right-hand side summarize the WaterLOGSY data for each compound, with the colored patches showing the relative degrees of burial of different parts of each molecule in the presence of IAPP (key on right). The % indicates the reduction in intensity of a resonance upon IAPP binding and is shown for protons for which inversion is not observed. The * indicates additional signals that originate from IAPP itself when IAPP is present (red), rather than the small molecule.

To characterize the molecular basis of the interaction between canagliflozin and IAPP monomers, WaterLOGSY NMR experiments were performed.^37^ While T1ρ experiments report on the relaxation rates of the compound upon binding to a macromolecular target, resulting in a uniform signal decay across all protons if binding occurs,^36^ WaterLOGSY involves magnetization transfer from water to free and protein-bound compound, thereby reporting on the change in solvent accessibility of the individual protons within the compound due to binding to protein.^37,38^ Representative WaterLOGSY spectra for canagliflozin, YX-I-1, doxazosin, dapagliflozin, and paracetamol in the absence or presence of IAPP monomers are shown in Figure 5a–e. The results provide further evidence that canagliflozin binds IAPP monomers with strong changes in all protons of the ligand (Figure 5a). The peaks corresponding to the aromatic protons and methyl substituent of the central phenyl ring are inverted in the presence of IAPP, indicating that they are particularly buried from solvent in the bound state, whereas the other aliphatic protons are strongly attenuated without inversion, indicating that they are more solvent-exposed when IAPP-bound. Signal attenuation, but not inversion, was observed for aromatic and aliphatic protons of YX-I-1 (Figure 5b) and doxazosin in the presence of IAPP (Figure 5c), consistent with weaker binding or a different binding pose. Additionally, the uniform decay of signals across all protons of YX-I-1 and doxazosin in the presence of IAPP suggests that there is no preferential orientation of these compounds in the bound state. Dapagliflozin (Figure 5d), one of the closest analogs of canagliflozin in the screening set, showed a pattern of binding similar to canagliflozin, with greater attenuation of the aromatic protons compared to the aliphatic protons, but binding was weaker overall and inversion of the aromatic peaks did not occur. As expected, the negative control, paracetamol, showed no effect in the WaterLOGSY experiment (Figure 5e). These data suggest that the primary site of molecular recognition of canagliflozin for IAPP is via its aromatic rings, leaving the *C*-glucosyl more solvent-exposed (Figure 5e). This agrees with the observation that dapagliflozin, which is chemically similar to canagliflozin but has a single aromatic ring (4-ethoxyphenyl) in place of the biaryl (2-(4-fluorophenyl)thiophene) present in canagliflozin, has weaker binding (Figures 4d and 5d) and does not significantly inhibit IAPP fibril self-assembly (Figure 2a,b).

To map the residues in IAPP that are involved in canagliflozin binding, ^15^N-labeled C-terminally amidated IAPP was prepared by recombinant protein expression as described previously^30^ (Methods), and ^1^H–^15^N SOFAST HMQC spectra were acquired in the absence or presence of a 5-fold molar excess of the small molecule (Figure S12). No statistically significant chemical shift perturbations (CSPs) or changes in the peak intensities of IAPP resonances were observed in the presence of canagliflozin (Figure S12). Thus, there appears to be no single well-populated bound state, consistent with weak affinity and/or suggesting that the interaction involves a heterogeneous ensemble of individually weak binding modes. Highly disordered binding has been observed for several small molecule ligands of intrinsically disordered polypeptides (e.g., 10074-G5 binding to Aβ(1–42),^39^ fasudil to the C-terminal 20 residues of αSyn,^40^ EPI-002/7170 to a 56-residue segment of the human androgen receptor,^41^ or 5-fluoroindole to the viral NS5A-D2D3 protein domains^42^) and can cause strong effects in ligand-detected NMR experiments despite there being small-to-negligible CSPs in protein-detected NMR experiments.^42^

### Canagliflozin Binds to Nascent Amyloid Fibrils

**Figure 6:**
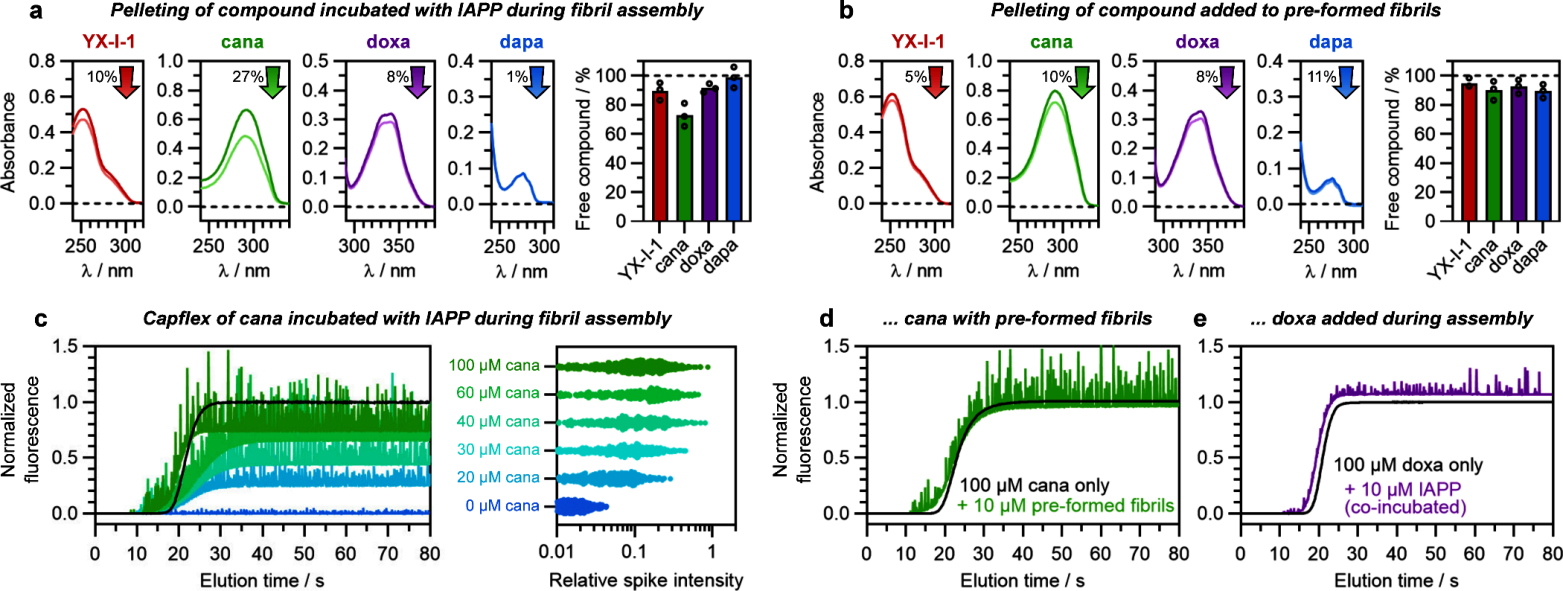
Canagliflozin interacts with IAPP amyloid fibrils. (a, b) Pelleting assays to quantify the remaining soluble concentration of compound after incubation (a) for 24 h with IAPP undergoing self-assembly or (b) for 30 min when added to preformed IAPP fibrils. In both cases, IAPP-compound mixtures were harvested, and the concentration of compound not adhering to fibrils was measured by acquiring reference-subtracted UV-absorbance spectra before (darker curves) or after (lighter curves) centrifugation to pellet fibrils (Methods). The percentage of free compound in solution after centrifugation is summarized in the bar chart on the right of each panel. Color corresponds to compound: red, YX-I-1; green, canagliflozin; purple, doxazosin; blue, dapagliflozin (negative control). Open circles in the bar charts represent individual biological repeats (3 each). (c) Microfluidic Capflex analysis of canagliflozin binding to IAPP during fibril assembly. Colored curves are Capflex elugrams of different concentrations (0/20/30/40/60/100 μM) of canagliflozin incubated with 10 μM IAPP for 24 h during fibril assembly, with the color scheme indicated in the figure. The black curve is 100 μM canagliflozin incubated without IAPP under the same conditions. Spikes reflect individual fibrils or clusters of fibrils, and increases in spike intensity result from fluorescent labeling of fibrils by canagliflozin, as summarized in scatter plots on the right. The final fluorescence plateau of each Capflex curve is proportional to the concentration of canagliflozin remaining in the soluble phase, i.e., not adhering to fibrils. The Capflex elugram with 30 μM canagliflozin has been omitted for clarity, but is included in the spike analysis on the right. Note that the Capflex elugram of IAPP alone has negligible change in fluorescence between the baseline (<15 s) and plateau (>30 s) due to the low intrinsic fluorescence of IAPP. (d) Capflex analysis with canagliflozin added after fibril assembly. (e) Capflex analysis of doxazosin incubated with 10 μM IAPP for 24 h during fibril assembly. The slight increase in fluorescent baseline with fibrils is not significant (within typical experimental error).

To investigate if the compounds also bind to amyloid fibrils, copelleting assays were performed. Fibrils were assembled in the presence of YX-I-1, canagliflozin, doxazosin, or dapagliflozin (used as a negative control), and the amount of small molecule bound to the fibrils in each case was quantified by pelleting the fibrils by centrifugation and measuring the concentration of soluble compound by absorbance spectroscopy (Methods). As shown in Figure 6a, ca. 27% of canagliflozin copelleted with fibrils, whereas copelleting of YX-I-1, doxazosin, and dapagliflozin was negligible (<10%). However, when the same concentration of canagliflozin was incubated (for 30 min) with fibrils formed in the absence of small molecule, only 10% was copelleted (Figure 6b). The enhanced binding of canagliflozin to coincubated versus preformed fibrils was judged to be significant by unpaired *t*-test (*p* = 0.042) and suggests that canagliflozin either binds to IAPP early in the self-assembly process and remains bound or diverts aggregation toward a structure that is more compatible with binding. Binding of YX-I-1, doxazosin, and dapagliflozin to preformed fibrils after self-assembly was negligible (Figure 6b).

We also took advantage of the intrinsic fluorescence of canagliflozin and doxazosin to assess fibril binding by Capflex assays (Figure S2b).^43^ Capflex is a fluorescence-based microfluidic technique that allows measurement of the free concentration of a solute and simultaneous detection of large (>1 μm) insoluble or phase-separated particles as discrete fluorescence spikes (Methods). Capflex was initially developed to screen for liquid–liquid phase separation,^43^ but has also been used to detect amyloid fibrils labeled with ThT^43^ or fluorescent peptides (“FibrilPaint”^44^). To confirm that canagliflozin associates with amyloid fibrils during assembly, we took advantage of the fact that IAPP only has weak intrinsic fluorescence (from Tyr37), whereas canagliflozin is ca. 30- and doxazosin is ca. 450 times more fluorescent than IAPP at the wavelengths used in the Capflex assay (Methods), respectively. This enabled us to use Capflex to detect binding of these molecules to fibrils when added during or after fibril assembly, by quantifying fluorescent spikes in the Capflex experiments. Co-incubation of canagliflozin with IAPP during fibril growth resulted in the appearance of large fluorescence spikes (Figure 6c), which are approximately 10 times larger than those seen for IAPP alone, indicating that canagliflozin binds to and fluorescently labels fibrils, although the exact degree of labeling cannot be inferred from spike intensity due to the likelihood of quenching effects. In addition, there was a significant (ca. 26%) reduction in the plateau fluorescence, indicating a loss of canagliflozin from the solution, most likely due to binding to fibrils. When added to preformed fibrils, spike formation was observed, but the loss of canagliflozin from solution was reduced, indicating a lesser degree of binding (Figure 6d). When doxazosin was coincubated with IAPP during self-assembly, a smaller increase in spike intensity was observed, without significant loss of the compound from solution, indicating that binding of doxazosin to IAPP fibrils is weaker or has a much lower stoichiometry than for canagliflozin (Figure 6d,e). We note that low-stoichiometry binding of doxazosin to fibrils could provide a possible mechanism by which doxazosin could interfere with ThT fluorescence and reduce the fluorescence intensity end point (Figure S8a). This effect is distinct from its inhibition of primary nucleation (Figure 3), which requires interactions with fibrils (or other intermediates) at an earlier stage of structural development.

### YX-I-1 and Canagliflozin Change the Observed IAPP Fibril Structures

**Figure 7:**
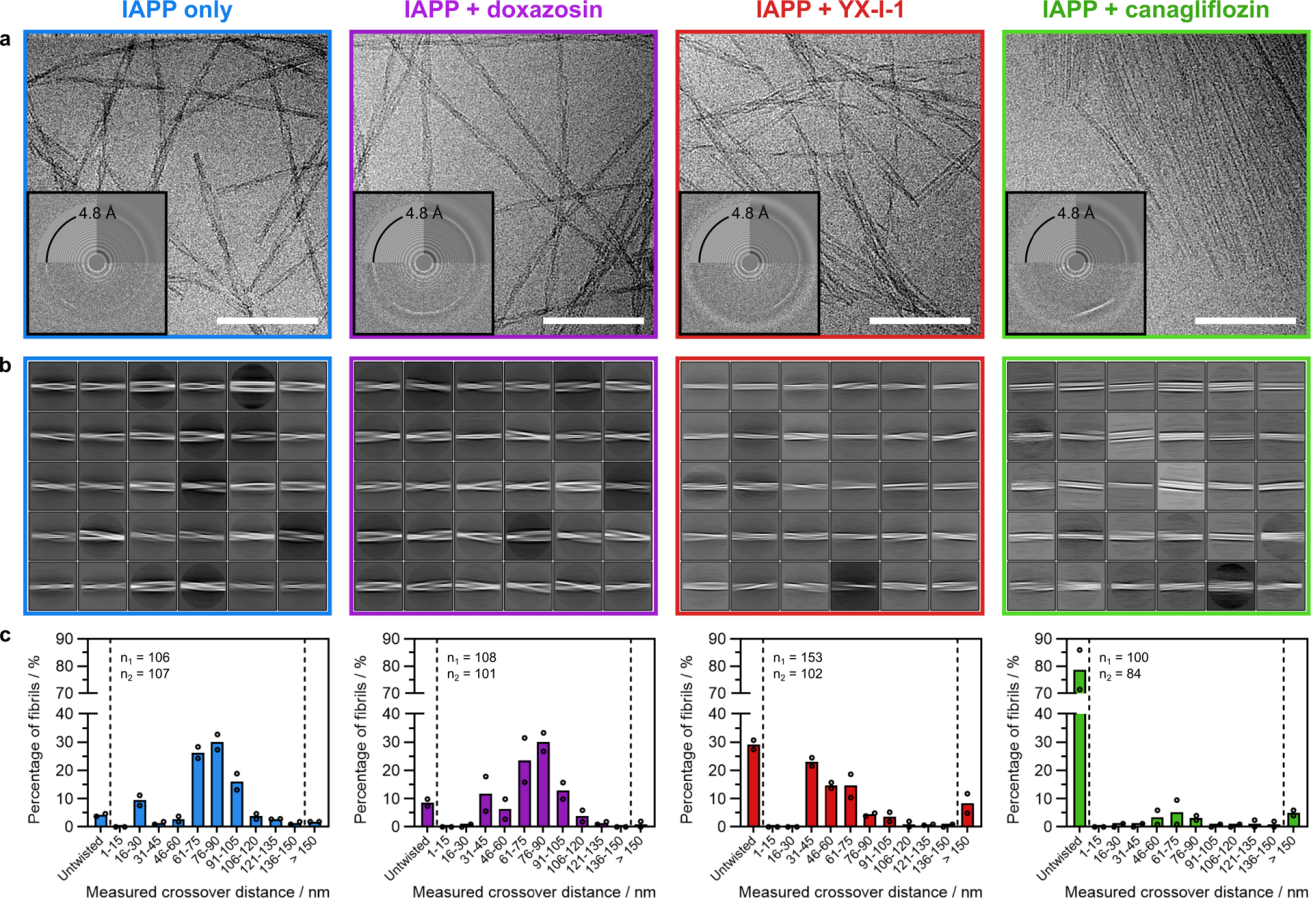
Cryo-EM of IAPP self-assembly reactions shows that YX-I-1 and canagliflozin alter the morphology of the amyloid formed, whereas doxazosin does not. (a) Representative cryo-EM micrographs of IAPP self-assembly reactions after 2 weeks in the presence of: no compound, doxazosin, YX-I-1, or canagliflozin. Scale bar = 100 nm. Individual twisting amyloid fibrils can be seen for the control, doxazosin, and YX-I-1 reactions, whereas samples with canagliflozin mostly contain sheet-like fibrillar material, infrequently interspersed with single fibrils. Corresponding power spectra (inset: top left corner is the estimated CTF, top right corner is the radially averaged raw data, and lower half is the raw spectrum itself) reveal that all four reactions have clear 4.8 Å peaks characteristic of cross-β amyloid fibrils. (b) 30 most populated 2D class averages from processing all fibril segments in each of the four conditions, showing that fibrils grown with doxazosin have similar morphology to the control, whereas those grown with YX-I-1 and canagliflozin have distinct morphologies. (c) Plots summarizing the fibril crossover distances of ∼100 individual fibrils measured directly from cryo-EM micrographs of two replicate reactions for each sample. Each reaction is represented by dots with the average plotted as bars, and the number of fibrils measured is indicated by *n*_1,2_.

Finally, we investigated whether the presence of canagliflozin, YX-I-1, or doxazosin during fibril assembly affects the structure of amyloid fibrils formed or whether the compounds slow fibrillization without affecting the final products. Cryo-EM was performed on fibrils assembled from 30 μM IAPP in the same buffer used for kinetic assays (160 mM ammonium acetate, pH 7.4, 1% v/v DMSO), in the absence (4 replicates) or presence (2 replicates each) of 50 μM YX-I-1, canagliflozin, or doxazosin (Methods). Fibrils were imaged after 2 weeks, at which point fibril formation was at a steady state, as judged by analyzing aliquots with ThT (Methods). As shown in Figure 7a,b, fibrils with a similar morphology were observed for the control (no small molecule) and doxazosin-containing samples, with the majority having a twisted ribbon morphology and crossover distances of 60–100 nm (Figure 7c). In the presence of YX-I-1, differences in fibril morphology were observed, with most (∼50%) fibrils having a shorter crossover distance of 35–75 nm, although there was also an increase in the proportion of fibrils with long crossovers (>150 nm), or no discernible twist (Figures 7a–c and S13). Most strikingly, canagliflozin induced a profound change in fibril morphology, inducing the formation of large, striated sheet-like assemblies, although some linear, mostly untwisted amyloid fibrils were also observed (Figures 7a–c and S13). As seen for typical amyloid fibrils, the power spectrum of the sheets grown in the presence of canagliflozin had a strong 4.8 Å signal, which aligned with the axis of the striations (Figure 7a, inset), supporting a β-sheet composition and consistent with the ThT fluorescence observed in kinetic assays. Similarly, fibrils formed in the absence of compound, or with YX-I-1 or doxazosin, also resulted in a clear 4.8 Å signal, consistent with amyloid formation (Figure 7a, insets).

**Figure 8:**
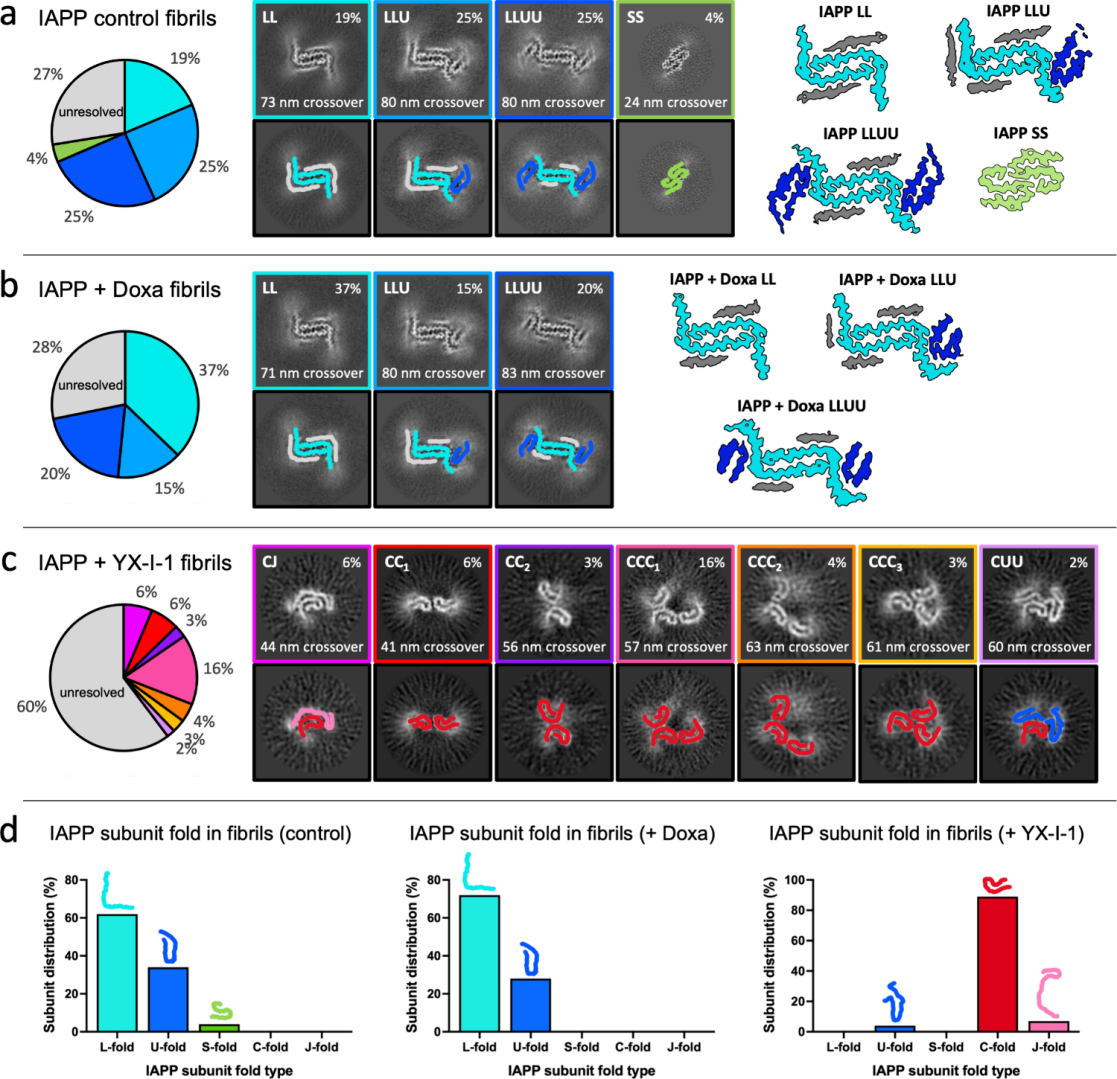
IAPP fibrils grown with YX-I-1 are composed of different subunit folds compared to the control and doxazosin-grown fibrils. Identified fibril polymorphs/structures after helical processing of fibril segments for (a) IAPP-only control, (b) IAPP + doxazosin, and (c) IAPP + YX-I-1 cryo-EM data sets. No fibril structures could be identified from the largely untwisted fibrils in the IAPP + canagliflozin cryo-EM data set. For each, a pie chart depicts the distribution of the full data set, with segments that could not be classified into a resolvable polymorph labeled as “unresolved”. Central z-slices of 3D class averages are shown for each identified polymorph (with colored box outlines relating to the pie chart) and replicated beneath with traced IAPP subunit backbone ribbons colored according to the subunit fold (black box outline). Where high-resolution structures were determined (gold-standard resolutions of 3.0–3.4 Å), flattened electron density maps are shown, colored according to the IAPP subunit fold. (d) Bar charts depict the distribution of IAPP subunit folds visualized within each population of fibril polymorphs (excluding unresolved fibril segments). Altogether, it is clear that fibril growth with YX-I-1 completely changed the IAPP subunit fold within the resolved fibril structures, whereas doxazosin did not.

Datasets from all four replicates of the compound-free control and one replicate with each compound were processed further. Following helical reconstruction (Figure S14), multiple distinct IAPP fibril polymorphs were resolved, revealing further detail on the apparent morphological differences between the samples. The majority of fibrils formed in the control (69%, Figures 8a and S15a–c) and doxazosin-treated (72%, Figure 8b) samples were classified into three related fibril structures (named LL, LLU, and LLUU, respectively), which have a shared structural core consisting of a pair of protofilaments related by a 2_1_-screw symmetry. Each protofilament is formed from a single stack of L-shaped IAPP subunits, with straight β-strand segments encompassing ^13^ANFLVHSSNNF^23^ and ^25^AILSSTNVGSNTY^37^-NH_2_, hinging on a turn at Gly24 (Figure S15d,e). The structure of this L-shaped subunit is distinct from the L-shaped subunit previously observed for the 2PF^L^ structure of the IAPP familial S20G variant^22^ (Figure S15f). In addition, further flanking subunits presenting U-shaped IAPP subunit conformations and ambiguous β-strands were observed adhering to these two β-sheets, with the combinations of these flanking subunits differing to generate the three individual polymorphs LL, LLU, and LLUU (Figures 8a,b and S16a,b). The well-known 2PF^S^ polymorph of wild-type IAPP fibrils^45−47^ was also found in the control samples, but was present at low levels (4%) in the conditions used here. Interestingly, 2PF^S^ was not found in the doxazosin-containing data set, consistent with the absence of fibrils with a ∼ 25 nm crossover in both of the replicate reactions imaged by cryo-EM (Figure 7b).

In the presence of YX-I-1, there was a striking, complete change in the population of fibril polymorphs, with none of the observed fibrils resembling any of the structures seen in the compound-free control or with doxazosin. The fibrils were still polymorphic but to a greater extent than in the control or with doxazosin. In all, seven unique fibril architectures were identified with YX-I-1, accounting for ∼40% of the total segments, with the remaining ∼60% having no observable crossover or a high degree of heterogeneity that prevented structure determination (Figure 8c). Due to the extreme diversity of the data set, the polymorphs formed in the presence of YX-I-1 could not be solved to high resolution, but cross sections of the classified maps showed that they consist of different arrangements of a shared C-shaped subunit, whose topology resembles (but structurally may be distinct from) the C-shaped subunit found previously in polymorphic IAPP S20G 2PF^C^ fibrils.^22^ In fact, one of the YX-I-1 polymorphs, which we term CC_1_, bears a remarkably similar architecture to the S20G 2PF^C^ structure from our previous study,^22^ with a similar crossover of ∼41 nm. Including the 60% unresolved segments in this data set, helical reconstruction starting with the 2PF^S^ or L-shaped structures from the control and doxazosin-containing samples failed to converge on those structures (Figure S14), indicating that those fibrils do not form in the presence of YX-I-1. Thus, out of the fibril structures that were identified in each data set, YX-I-1 fibrils consisted of 89% C-fold subunits, whereas this subunit fold was absent from fibrils grown in the control or with doxazosin. By contrast, the control and doxazosin-treated samples consisted of L-fold (62% or 72%, respectively), U-fold (34% or 28%), and S-fold (4% or 0%) subunits (Figure 8d). Finally, no twisted fibril structures could be resolved in the data set of IAPP with canagliflozin. Attempts to solve the structure(s) of these assemblies using the fibril structures identified here for wild-type IAPP and previously for IAPP S20G^22^ as starting templates were also unsuccessful. Together, the cryo-EM data analysis demonstrates that YX-I-1 and canagliflozin direct IAPP assembly to amyloid architectures that are different from each other and those observed in the control or with doxazosin.

Lastly, we investigated whether the change in amyloid architecture in the presence of YX-I-1 and canagliflozin results in a change in seeding activity. Fibrils were prepared by incubating 10 μM IAPP for 24 h in 160 mM ammonium acetate (pH 7.4) with 1% (v/v) DMSO at 30 °C, in the absence or presence of 50 μM doxazosin, YX-I-1, or canagliflozin, before being extracted by centrifugation and used to seed new self-assembly reactions in the absence of compound (Methods). In agreement with the results shown in Figures 7 and 8, doxazosin-grown fibrils had the same seeding potency as control fibrils grown without compound, whereas YX-I-1 and canagliflozin-grown samples had enhanced potency (Figure S17). A compound-induced change in fibril structure could directly affect the seeding potency by altering the rate of elongation or secondary nucleation or could indirectly alter the seeding potency by affecting processes such as flocculation, which can reduce the activity of fibril seeds in the plateau phase. As a result, it is not possible to directly relate the changes in seeding potency to the effects on κ in the growth phase (Figure S8b). Nonetheless, these data clearly show that the compound-induced changes in amyloid structure result in a change in activity, in this case, the seeding potency.

## Discussion

Here, we have investigated whether regulator-approved small molecule drugs can be repurposed as inhibitors of IAPP amyloid formation, building upon our previous work that identified YX-I-1 as a lead.^26^ Virtual screening, ThT assays, and biophysical characterization successfully identified two widely used FDA-approved drugs, canagliflozin and doxazosin, as inhibitors of IAPP amyloid formation. Canagliflozin, currently used as a third-line type-2 diabetes medication with an intended mode of action (SGLT2 inhibitor) unrelated to islet amyloid, had the strongest inhibitory effect of all the molecules tested. Whether treatment with canagliflozin provides additional clinically relevant benefits through an effect on IAPP fibrillization is unknown, although canagliflozin has been shown to improve pancreatic β-cell function via an uncharacterized SGLT2-independent mechanism.^48,49^ On the other hand, canagliflozin may currently be given too late in disease progression to take advantage of any therapeutic benefits by inhibiting IAPP amyloid formation. In addition, *ex vivo* structure determination of IAPP fibril deposits and clinical/animal studies will be required to determine whether canagliflozin treatment results in a change in IAPP fibril polymorphism in patients and whether this affects disease outcomes. Doxazosin, on the other hand, is not currently used as a type-2 diabetes medication, but is widely used for conditions such as benign prostatic hyperplasia, and may also affect IAPP aggregation if administered to diabetic people with these conditions. As canagliflozin and doxazosin are both widely used drugs, and could be rapidly repurposed as amyloid-targeting type-2 diabetes treatments, the clinical effects of any current interactions with islet amyloid and the effects of early treatment in pre- or early stage diabetic individuals should be investigated as a matter of urgency.

Canagliflozin also provides a promising lead for the future development of compounds that are more potent inhibitors of IAPP amyloid formation and could be specifically optimized for this mode of action. Although a full structure–activity relationship (SAR) will require further work in the future, our study nonetheless reveals tantalizing clues as to the structural basis of canagliflozin’s activity. We identified canagliflozin through its structural similarity to YX-I-1, particularly the presence of a shared tetrahydropyran moiety, which our preliminary SAR (Figure S1) had suggested was important for inhibition of IAPP assembly into amyloid, and its high ComboScore in the ROCS analysis. However, our subsequent WaterLOGSY experiments (Figure 5) suggested that canagliflozin has a distinct binding mode, with the aromatic rings experiencing a high degree of burial from solvent when in complex with IAPP monomer and the *C*-glucosyl more exposed. This binding is reminiscent of that of dapagliflozin (Figure 5d), a noninhibitory compound with a much weaker affinity for IAPP (Figure 4), but binding by canagliflozin appears to be enhanced by the presence of a more extensive aromatic ring system. The difference in binding mode between the flozins and YX-I-1 may lie in the fact that the *C*-glucosyl moiety is much more hydrophilic than the equivalent tetrahydropyran-containing part of YX-I-1 (as well as the equivalent in doxazosin). Future SAR studies should evaluate whether the *C*-glucosyl of canagliflozin is required for activity, as elimination or substitution of this region would also be expected to abolish SGLT2 binding, allowing for optimization toward a specifically amyloid-targeting mode of action. In addition, given their importance in monomer binding, substitutions on the aromatic rings should be investigated, as they may be key to inhibiting or structurally steering IAPP amyloid formation.

Our kinetic analysis showed that canagliflozin most strongly inhibits primary nucleation of wild-type IAPP amyloid fibrils, similar to YX-I-1.^26^ In addition, canagliflozin causes a weak acceleration of the secondary pathway, which could arise from a change in fibril structure. Primary nucleation is challenging to study directly because it is an activated process involving a short-lived, unstable transition state (i.e., the critical nucleus). Experimental studies of primary nucleation are therefore inevitably restricted to species immediately before or after this transition. Here, we have demonstrated that YX-I-1, canagliflozin, and doxazosin bind IAPP monomers, whereas noninhibitory compounds from the screen do not. This suggests that monomer binding is related to inhibition, but monomer binding alone is too weak to rationalize the effectiveness of the compounds in retarding amyloid formation. We also showed using pelleting and Capflex assays that canagliflozin binds IAPP fibrils and that the binding is enhanced when canagliflozin is present during fibril growth, implying that it binds one or more species formed at an intermediate stage of assembly. These observations suggest that inhibition of primary nucleation rests on the ability of these compounds to bind IAPP throughout the nucleation process, allowing them to sculpt the energy landscape of nucleation in a manner that increases the nucleation barrier and slows the formation of the naïve (i.e., without compound) fibril fold(s). This sculpting of the energy landscape also provides a means for compounds to steer assembly toward new fibril architectures. Previous experimental and computational studies of early IAPP fibril development have suggested a spreading of cross-β structure from the central ^20^SNNFGAILSS^29^ region to the rest of the sequence,^50^ suggesting that there may be a common structural checkpoint that YX-I-1 and canagliflozin target. However, the precise molecular basis of this inhibition remains unclear and may well differ between each compound, given that YX-I-1, canagliflozin, and doxazosin differ in chemical structure, inhibition kinetics, and their effects on IAPP fibril structure.

Our data also suggest a relationship between the strength of kinetic inhibition and the extent of the change in polymorphism, with doxazosin having a limited effect on the rate of primary nucleation and little effect on polymorphism, whereas YX-I-1 and canagliflozin strongly inhibit primary nucleation and cause a profound change in fibril polymorphism. One possible interpretation of this finding is that primary nucleation acts as a branch point where a decision is made between potentially many different families of fibril polymorphs, and inhibitors of primary nucleation are able to close off some, but not necessarily all, avenues of fibril development and/or open up pathways to new fibril folds. Interestingly in this regard, we recently reported that IAPP S20G evolves through a sequence of different polymorphs during amyloid formation, with the early fibril structures being more kinetically accessible, but without major differences in thermodynamic stability compared to their later counterparts.^22^ A kinetic evolution of fibril folds has also been observed by cryo-EM for wild-type IAPP amyloid^22^ and recombinant tau(297–391).^23^ These early fibril species may be ideal targets for small molecule binding to change the course of amyloid assembly by differential stabilization or alteration of their surface properties. Further cryo-EM investigation tracking the detailed course of IAPP self-assembly in the presence of canagliflozin and YX-I-1 will be needed to determine precisely how these molecules steer fibril development and bring about such wholesale shifts in fibril polymorphism.

In summary, the results presented demonstrate that small molecules that inhibit the microscopic steps of amyloid self-assembly can sculpt the energy landscape of nucleation, allowing them to profoundly alter the structure of the resulting fibril products. Thus, the effects of inhibitors can last well beyond the lag and growth phases of amyloid formation. Future characterization of the compound-IAPP binding sites and the requirements for sustained interaction as nucleation progress may allow for the design of more potent agents capable of steering the structural evolution of fibrils down a different path. The ability to control fibril polymorphism at the molecular level using small molecules will also provide new opportunities to better understand the relationship between fibril structure and cellular (dys)function. Such tools may be powerful weapons in the fight against diseases involving amyloid formation.

## Methods

### Virtual Screening

Substructure searches were performed for tetrahydropyran-containing compounds in commercially available compound libraries (Figure 1a). The importance of the tetrahydropyran-containing portion of YX-I-1 was suggested by preliminary SAR experiments showing that readily available analogs that lack that portion of the molecule do not inhibit IAPP aggregation (Figure S1), although a role for the substituents cannot be excluded, and the rest of YX-I-1 may also be important for activity. Tetrahydropyran-containing compounds that were polyphenols were excluded. All identified tetrahydropyran-containing compounds were also part of the FDA-approved drug library (SelleckChem, 3008 compounds) used in ligand-based similarity screening using ROCS, or were otherwise regulator-approved. In total, 14 compounds were identified by these searches (Table S1).

Ligand-based similarity screening was performed using ROCS (Rapid Overlay of Chemical Structures) (OpenEye Scientific). Separate ROCS searches were performed using either of the two enantiomers of YX-I-1 as the query molecule (Figure 1b). In each case, YX-I-1 was minimized using the default settings within the LigPrep tool in Maestro (Schrödinger) and then used as the template for a ROCS search of the FDA-approved drug library (SelleckChem, 3008 compounds), prepared, and energy-minimized in the same way as for the query ligand. The degree of structural similarity was quantified using the ROCS combo score, which is the sum of two separate Tanimoto scores measuring the degree of overlap of the “shape” and “color” of the molecules. While the “shape” score quantifies volume overlap, the “color” score considers alignment of chemical features, specifically charge, rings, hydrophobes, and hydrogen bond donors/acceptors. The top 10 hits for either enantiomer, ranked by combo score, were combined to give a preliminary set of 17 compounds after accounting for three overlaps. Two of these were excluded before purchase: benzonatate, as it is sold with PEGylation of varying length; and fursultiamine, which has a disulfide bond. This resulted in a final combined set of 15 ROCS hits (Tables S2 and S3).

As 10/14 of the compounds identified by substructure searches were also in the FDA-approved drug library used for ROCS searches, we examined the combo scores of these compounds against either enantiomer of YX-I-1 and their ranking relative to other compounds in the library (Table S1). None of these compounds was ranked in the top 10 for either enantiomer, but a high proportion (8/10) were ranked in the top 10% (ie. top 300) for enantiomer 1 and/or 2, validating the structural similarity of these compounds to YX-I-1. On average, the compounds had higher similarity (combo score) to enantiomer 1 (0.689 ± 0.060, *n* = 10) than they did to enantiomer 2 (0.600 ± 0.035, *n* = 10) and this was significant at the *p* < 0.05 level (*p* = 0.0028 by paired *t*-test). Examination of ROCS alignments (Figure 1c) revealed that, among the 5 compounds that were ranked in the top 10% of the FDA-approved drug library for similarity to enantiomer 1 alone (daidzin #22, canagliflozin #45, liquiritin #75, polydatin #98, dapagliflozin #246), the tetrahydropyran ring (as part of *C*- or *O*-glucosyl) within the molecule overlapped with the tetrahydropyran of YX-I-1 in all cases except for liquiritin.

The 14 compounds from substructure searches and 15 compounds from ligand-based similarity screening were combined to give a set of 29 compounds. The druglikeness of these compounds was examined by collating their basic physicochemical properties and any Lipinski violations as listed on PubChem^51^ (Table S4). All 29 compounds were then taken forward to the solubility screening.

### Stock Preparation and Quality Control of Small Molecules

Small molecules were purchased at >98% purity (Cambridge Bioscience, Clinisciences Ltd.), dissolved to 25 mM in DMSO-*d*_6_, and stored at −20 °C. The molecular weight was confirmed in-house by liquid chromatography mass spectrometry (LC-MS).

Absorbance spectra (235–750 nm) of each compound were acquired to identify spectral overlaps that might affect assays (e.g., levosimendan with ThT, Figure S6c–e) and to identify any light scattering caused by solubility issues. Initially, absorbance spectroscopy was performed with 50 μM compound in 160 mM ammonium acetate (pH 7.4) with 1% v/v DMSO (the intended buffer for downstream assays), using a UV-1800 spectrophotometer (Shimadzu) with a quartz cuvette. Possible light scattering by three compounds (abiraterone acetate, fluralaner, and rafoxanide) was identified as significant attenuation in the 400–750 nm range that did not correspond to a clear absorption peak (Figure S3) and was confirmed by acquiring additional spectra of (i) the compounds at varying concentrations from 5 to 50 μM and (ii) the supernatant of 50 μM compound that had been centrifuged for 30 min at 16,300 *g*. For these three compounds, optical density across wavelengths was not proportional to the nominal concentration of the compound, consistent with light scattering but not absorption, and optical density in the 400–750 nm range was eliminated completely by pelleting, confirming that it resulted from scattering by insoluble particles. Fenbufen, used as a soluble control in the confirmatory experiments, had no attenuation in the 400–750 nm range, and its absorption peak around 284 nm was proportional to the concentration and unaffected by pelleting. Spectra were analyzed in UVProbe (Shimadzu), Spectragryph v1.2.16.1,^52^ and GraphPad Prism 10.

Solubility screening was also carried out by Capflex^43^ (Figures S2 and S3). Solutions of 50 μM of each compound were prepared in 160 mM ammonium acetate (pH 7.4) with 1% v/v DMSO, in a clear 96-well pressure plate with a plate seal (FidaBio). The plate was placed in the autosampler of a Fida-1 instrument, with the autosampler and capillary both equilibrated to 30 °C. Capflex runs were performed as described in the “Capflex and TDA” section, with two independent biological repeats per compound. Capflex elugrams were analyzed in the Fida analysis software supplied with the instrument and GraphPad Prism 10. For compound solubility screening, the purpose of Capflex was to test for intrinsic fluorescence spikes that occur when large aggregates of insoluble compounds pass the detector. In total, 23/32 compounds (out of a set including the 29 screening compounds, YX-I-1, paracetamol, and EGCG) had sufficient intrinsic fluorescence that spikes should be detectable if sufficiently large insoluble particles had formed, whereas the solubility of the remaining 9/32 compounds could not be assessed by Capflex. Of the compounds whose solubility could be assessed, 19 had spike-free Capflex elugrams and were passed, and 4 had spikes indicating poor solubility.

Compounds that showed sufficient fluorescence and solubility by Capflex were subsequently analyzed by Taylor dispersion analysis (TDA)^53,54^ (Figures S2 and S4). TDA was performed to check that the hydrodynamic radius (*R*_h_) of the soluble compounds was as expected, i.e., that oligomerization was not occurring. Solutions of 50 μM compound were prepared in 160 mM ammonium acetate (pH 7.4) with 1% v/v DMSO, in 100 μL volumes in glass pressure vials with inserts (FidaBio). The autosampler and capillary temperatures were set to 30 °C. TDA runs were performed and analyzed as described in the section on “Capflex and TDA” section. *R*_h_ measurements could be obtained for 17/19 compounds, whereas 2/19 had insufficient intrinsic fluorescence. All *R*_h_ measurements were compatible with small molecules (<0.7 nm), and a positive correlation (Pearson *r* = 0.7509) was observed between molecular weight and measured *R*_h_. The lack of strong outliers or *R*_h_ measurements above 0.7 nm suggested that significant oligomerization of the small molecules was not occurring.

Solubility screening data were collated, and any compounds that failed either the absorbance spectroscopy or Capflex steps (as none failed TDA) were excluded from subsequent screening for inhibition of IAPP self-assembly. In total, 24/29 compounds identified by virtual screening were passed as well as 3/3 of the additional compounds that were subjected to solubility screening (YX-I-1, paracetamol, and EGCG). We note that the compounds that failed solubility screening had significantly higher ClogP values (average 5.4 ± 0.5) than those that passed (2.2 ± 0.2), at the 0.05 level of significance (*p* < 0.0001 by unpaired one-tailed *t*-test).

### IAPP Synthesis and Purification

Wild-type IAPP and the S20G variant were chemically synthesized complete with C-terminal amidation, and the Cys2-Cys7 disulfide bond was formed after synthesis. Synthesis and purification protocols are based on previous protocols.^26,55,56^ Synthesis was performed on a Liberty Blue automated microwave peptide synthesizer (CEM Microwave Technology) on a 0.25 mmol scale, with PAL-NovaSyn TG resin (Novabiochem, Merck), 9-fluorenylmethyloxycarbonyl (Fmoc)-protected amino acids, hexafluorophosphate benzotriazole tetramethyl uronium (HBTU) (Merck) as activator, and *N*,*N*-diisopropylethylamine (Sigma) as base. For wild-type IAPP, three pseudoproline dipeptides [Fmoc-Ala-Thr(psiMe,Mepro)–OH, Fmoc-Ser(*t*Bu)-Ser(psiMe,Mepro)–OH, and Fmoc-Leu-Ser(psiMe,Mepro)–OH, Merck] were coupled in place of Ala8-Thr9, Ser19-Ser20, and Leu27-Ser28. For S20G, the Fmoc-Ser(*t*Bu)-Ser(psiMe,Mepro)–OH dipeptide was replaced with standard coupling of Ser19 and Gly20. All of the residues were double-coupled. After synthesis, the resin was washed with dimethylformamide (DMF), dichloromethane (DCM), and diethyl ether, and the peptide was cleaved from the resin in a cocktail of 92.5% v/v trifluoroacetic acid (TFA), 2.5% v/v 3,6-dioxa-1,8-octanedithiol (DODT), 2.5% v/v triisopropylsilane (TIPS), and 2.5% v/v water. The cleavage mixture was left for 4 h on a rotator, then collected, and concentrated under a nitrogen stream. Crude peptide was precipitated in cold diethyl ether, followed by three washes with the same solvent, and then resolubilized in 1:1 acetonitrile/water and lyophilized. Peptide was redissolved in 1:1 DMSO/water and left for 36 h to allow DMSO-induced formation of the Cys2-Cys7 disulfide bond, before purification by two rounds of mass-directed HPLC in water/acetonitrile with 0.1% v/v formic acid as a modifier, lyophilizing and resolubilizing in 1:1 DMSO/water in between. After the second round, the peptide was lyophilized, redissolved in 0.1% v/v formic acid aqueous solution, and quantified by UV-absorbance spectroscopy using an extinction coefficient of 1615 M^–1^ cm^–1^ at 280 nm (determined using Expasy^57^). The mass of the purified IAPP (wild-type 3902.9 Da; S20G 3872.9 Da) was determined by high-resolution mass spectrometry (HR-MS), confirming the presence of the Cys2-Cys7 disulfide and C-terminal amide (expected masses 3903.3 and 3873.3, respectively), and the peptide was then aliquoted, lyophilized, and stored at −20 °C.

Size exclusion chromatography (SEC) was performed prior to IAPP self-assembly experiments to isolate the monomeric peptide. A HiPrep (16/60) Sephacryl S-100 HR column was pre-equilibrated in a 1.02× stock of the intended experimental buffer (see “Experimental Buffers for IAPP”), at 5 °C. Sephacryl resin reduces secondary interactions of IAPP with the column matrix, compared to Superdex.^30^ Aliquots of lyophilized IAPP were thawed, dissolved to 10 mg/mL in DMSO with gentle agitation for 5 min, and diluted 10 times into ice-cold SEC running buffer. This was mixed, centrifuged for 5 min at 16,300 *g*, and injected into the column. Protein was eluted at 0.4 mL/min and the peak at ca. 84 mL, previously identified as monomer^30^ and further confirmed by HR-MS and FIDA, was collected on ice, quantified by UV absorbance spectroscopy, and combined with additional 1.02× buffer stock, DMSO (with or without compound), and 100× ThT stock where relevant.

### Experimental Buffers for IAPP

Except where otherwise stated, experiments were performed in 160 mM ammonium acetate (Sigma), adjusted to pH 7.4 with ammonia solution, and filtered to 0.22 μm before use. Ammonium acetate was chosen to maximize compatibility with downstream experiments, match the ionic strength (ca. 160 mM) of interstitial fluid, and because acetate is monovalent and has an intermediate ionic radius similar to the most abundant physiological anions, chloride and bicarbonate.^58^ The latter criteria are important as IAPP is sensitive to ionic strength and specific interactions with anions.^59^ Despite the distance from the p*K*_a_ of ammonium (9.1 at 30 °C^60^), the high concentration of 160 mM ammonium acetate gives it a reasonable buffer capacity β = d*C*/d*p*H, i.e., the concentration of added strong acid/base versus the resulting change in pH. At 30 °C, 160 mM ammonium acetate has β = 7.2 mM, compared to 4–5 mM for typical phosphate-buffered saline (PBS) formulations. For NMR experiments, we used 25 mM sodium phosphate (pH 6.8) for its enhanced buffer capacity (14 mM), lower pH, which reduced exchange of protons, and lower ionic strength (45 mM), which extended the lifetime of monomeric IAPP to cover NMR time scales. In all cases, buffers also contained 1% v/v DMSO to act as a vehicle for small molecules.

### Capflex and TDA

The principles of Capflex and Taylor dispersion analysis (TDA) are summarized in Figure S2. In brief, both are microfluidic instruments, with Capflex involving flowing larger quantities of sample (10–20 μL) continuously past a detector to measure the soluble concentration and abundance of insoluble particles and TDA involving flowing a small (∼50 nL) plug down a capillary to measure its dispersion and thus calculate the hydrodynamic radius (*R*_*h*_) of constituents. Capflex and TDA were carried out on a Fida-1 instrument (FidaBio) with a 75 μm x 1 m capillary, washed with 1 M NaOH, and coated with HS reagent (FidaBio). In both types of experiment, elution was monitored by intrinsic fluorescence (excitation 275 nm, emission 300–450 nm), with a default photomultiplier tube (PMT) voltage of 570 V, although 500 V was used for Capflex of doxazosin-containing samples to avoid saturating the detector, as doxazosin has a high intrinsic fluorescence. Capflex and TDA data were analyzed in the Fida analysis software supplied with the instrument, Microsoft Excel 2019, and GraphPad Prism 10.

For each Capflex run, the capillary was flushed with blank (ie. buffer with DMSO but no compound) for 120 s at 3500 mbar, and the sample was then injected/eluted continuously through the capillary for 80 s at 2000 mbar (with an additional 10 s tail to ensure that pressure remained stable for the 80 s that were analyzed). Separate procedures were followed for analysis of Capflex data, depending on whether experiments were for compound quality control or measuring fibril binding, as detailed in the relevant sections.

Each TDA run had three steps: (i) the capillary was flushed/equilibrated with blank (ie. buffer with DMSO but no compound) for 120 s at 3500 mbar; (ii) a plug of sample was injected for 10 s at 50 mbar; and (iii) the sample was eluted by application of further blank for 180 s at 400 mbar. The diffusion coefficient *D* was determined by fitting the Taylor dispersion equations,^53^
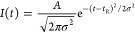
1
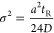
2where *I*(*t*) is the baseline-subtracted fluorescence signal, *A* is the peak area, *t* is the measurement time, *t*_R_ is the measurement time of the peak center, and *a* = 37.5 μm is the capillary radius. *A*, *t*_R*,*_ and *D* were fitted parameters, and fitting was performed in Fida analysis software (FidaBio). *R*_h_ was determined using the Stokes–Einstein relation,
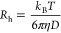
3where *k*_B_ is the Boltzmann constant, *T* = 303 K is the capillary temperature, and η = 0.797 mPa.s is the viscosity of the medium.

### Thioflavin T (ThT) Assays

Monomeric IAPP, isolated by SEC as described above (“IAPP synthesis and purification”), was prepared to the desired concentration in a final buffer of 160 mM ammonium acetate (pH 7.4) with 1% v/v DMSO and 20 μM ThT. The reaction mixture was pipetted into the wells (100 μL each) of a low-binding 96-well microplate (Corning 3881, NY), typically with 3–5 replicate wells per condition. Each plate also contained 3–5 blank wells with the same constituents, but no IAPP. The plate was sealed with an adhesive polyester film (Labstuff, UK) to restrict evaporation and incubated in a CLARIOstar plate reader (BMG Labtech, UK) at 30 °C without shaking. Fluorescence readings were taken every 5 min quiescently, with excitation at 440 nm and emission at 480 nm. Raw fluorescence intensities were baselined by subtracting the average fluorescence intensity of blank wells from the same experiment. Data were normalized by dividing the blank-subtracted fluorescence intensities by the maximum average blank-subtracted fluorescence intensity across replicate wells.

### Mathematical Analysis of Amyloid Self-Assembly Kinetics

Amyloid self-assembly kinetics were analyzed by fitting the normalized ThT fluorescence intensities to equations describing the conversion of free monomer to fibrils, as described below. For nucleated polymerization without secondary processes, which was included in our initial analysis of uninhibited IAPP self-assembly kinetics (Figure S5, Table S6), we used Oosawa’s exact solution,^61^
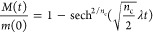
4where *M*(*t*) is the effective concentration of fibrillar IAPP monomers, *m*(0) is the initial free concentration of IAPP monomers (such that *M*(*t*)/*m*(0) is the proportion of monomer converted to fibril), and *n*_c_ is the effective reaction order of primary nucleation. The macroscopic rate parameter λ describes the rate at which monomer is converted to fibril due to the nucleated polymerization,
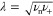
5Here, ν_n_ and ν_+_ are the normalized rates of primary nucleation and elongation, respectively, and are usually defined by the rate laws,^34,61^
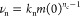
6
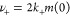
7where *k*_n_ and *k*_+_ are microscopic rate constants. For all other analyses, we used the recent equation for nucleated polymerization with secondary processes obtained by perturbative renormalization group analysis,^62^ although we adopt a simplified nomenclature in line with earlier work,^34,35,63^
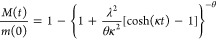
8Here, θ is a dimensionless parameter that determines the sensitivity of the secondary pathway to depletion of monomer in the late growth phase,^63^ and κ is an additional macroscopic rate parameter that describes the rate of the secondary pathway.^34^ The precise definition of κ depends on the dominant secondary process. If fragmentation dominates,^34^
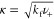
9where *k*_f_ is the first-order rate constant for fibril fragmentation. If secondary nucleation dominates,^35^
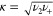
10where the normalized rate of secondary nucleation, ν_2_, has different definitions for single-step secondary nucleation,^35^
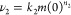
11and multistep secondary nucleation,^62^
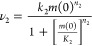
12where *k*_2_ is the microscopic rate constant, *n*_2_ is the effective reaction order, and *K*_2_ is the effective Michaelis constant for secondary nucleation.

In our analysis of IAPP self-assembly kinetics without inhibitors (Figure S5 and Table S6), we globally fitted the normalized ThT fluorescence intensities acquired at IAPP concentrations from 8 to 30 μM to equations reflecting 4 scenarios: no secondary processes (eqs 4–7), fragmentation-dominated (eqs 5–9), single-step secondary nucleation (eqs 5–8, 10, and 11), and multistep secondary nucleation (eqs 5–8, 10, and 12). Fit quality was quantified by Akaike’s corrected information criterion (AICc), and differences in AICc (ΔAICc) were calculated relative to the model with the lowest AICc. *R*^2^ values were also calculated. The fitted parameters and metrics describing fit quality are detailed in Table S6.

In our model comparison in Figure 3a–c, we performed global fitting using the model for nucleated polymerization with dominant secondary nucleation, eqs 5–8, 10, and 12. In each case, the rate of primary nucleation, secondary nucleation, or elongation was allowed to vary with inhibitor concentration, whereas the others were shared globally. We set θ = 0.323 in line with the results of the analysis in Table S6, although varying θ did not affect the outcome of the model comparison. Fit qualities were compared by AICc and ΔAICc, and the comparison of fits is provided in Table S8.

For our extraction of the macroscopic rate parameters in Figure 3f,g, we repeated the fitting on the same data using the same model (eqs 5–8, 10, and 12), but performed fitting at the level of λ and κ (rather than expressing these in terms of ν_n_, ν_2_, and ν_+_) and plotted the fitted values of λ and κ.

Kinetic analysis was performed in GraphPad Prism 10 using nonlinear least-squares regression.

### Quantitation of Soluble IAPP Monomer

To quantify final fibril yield (Figure 3e), the contents of reactions were extracted from plate wells or vials, pooled in volumes of 250 μL, and centrifuged for 30 min at 16,300 *g* to pellet aggregates. Taking care not to disturb the pellet, 200 μL of supernatant was then aspirated, supplemented with an equal volume of DMSO and 1% v/v TFA, incubated at 37 °C for 12 h with shaking to monomerize any nonpelleted material, and then stored at −80 °C. To quantify monomer disappearance at intermediate time points between the lag and plateau phases (Figure S9), the same procedure was followed, but the pooled volume was 200 μL, the centrifugation time was 15 min, and the volume of supernatant aspirated was 150 μL. The soluble monomer remaining in each sample was then quantified by analytical HPLC using a Nexera LC-40 (Shimadzu) with a Nucleosil 300 C4 column (5 μm, 250 × 4.6 mm) through a PEEK precolumn filter, with 0.1% v/v TFA solution as solvent A and acetonitrile +0.1% v/v TFA as solvent B. Samples were eluted with a 5–80% gradient of solvent B, operating at a flow rate of 1 mL/min. Autosampler vials were 300 μL PP insert vials with PTFE and aluminum lids (ThermoFisher). The IAPP monomer peak was detected by UV absorbance at 220 nm by using an SPD-M40 photodiode array detector as part of the instrument. Peak integration and analysis were performed in the LabSolutions software supplied with the instrument as well as GraphPad Prism 10.

### Surface Plasmon Resonance

The interaction between wild-type IAPP and small molecules was analyzed on a Biacore 1K+ instrument (Cytiva) at 30 °C. IAPP was N-terminally biotinylated by reaction with NHS-PEG_4_-biotin (Thermo Scientific), which labels either the α- or ε-amino group of Lys1 (the only lysine in IAPP). Labeling was carried out with 1 mM IAPP and 1 mM NHS-PEG_4_-biotin in 50 mM sodium phosphate (pH 7.4) with 50% (v/v) DMSO to ensure solubility. Single-labeled IAPP was purified by mass-directed HPLC (4376.5 Da), the purity was confirmed by liquid chromatography with HR-MS, and the peptide was quantified by UV-absorbance spectroscopy. Biotinylated IAPP was immobilized on a streptavidin-coated sensor chip (Cytiva) to a functionalization of 1350 RU, with an untreated flow cell used as the reference surface. Small molecule binding was analyzed in a running buffer of 160 mM ammonium acetate (pH 7.4) with 1% v/v DMSO, with equilibration for 9 s followed by an association phase of 60 s and dissociation phase of 90–120 s. Measurements were performed 3 times for each compound. Data were referenced and then blank-subtracted, using the same buffer with 1% v/v DMSO but no small molecule as a blank.

The relationship between canagliflozin concentration and the SPR plateau (Figure S11a) was determined by individually fitting each SPR trace with a biexponential model to the association phase (0–60 s),
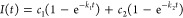
13where *k*_1_ and *k*_2_ are the rates, and *c*_1_ and *c*_2_ are the amplitudes of the two phases, so that the plateau response is *A*_1_ + *A*_2_. Global fitting of SPR traces with varying canagliflozin (Figure S11b) was performed by fitting a single-exponential association and dissociation model,
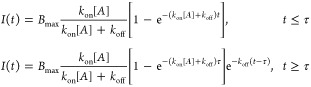
14where *B*_max_ is the response at complete saturation, *k*_on_ is the second-order association rate constant, [*A*] is the concentration of analyte, *k*_off_ is the first-order dissociation rate constant, and τ = 60 s is the time at which the dissociation phase started. Fitting with free *B*_max_, *k*_on_ and *k*_off_ gave arbitrarily high values of *B*_max_ and *K*_D_ = *k*_off_/*k*_on_, indicating that *K*_D_ is well above the maximum analyte concentration and cannot be determined. For final data presentation, we set *B*_max_ = 10^4^ RU, which gave *K*_D_ = 25 mM. All fitting of SPR data was performed in GraphPad Prism 10.

### NMR

The T1ρ and WaterLOGSY NMR experiments were performed with 100 μM ligand in the absence or presence of 20 μM unlabeled IAPP, in 25 mM sodium phosphate buffer (pH 6.8) with 1% v/v DMSO-*d*_6_ at 5 °C. Spectra were acquired by using a Bruker Avance III-HD 600 MHz spectrometer. T1ρ spectra were acquired with 512 scans per point (ns) and 200 ms spinlock pulse, whereas WaterLOGSY spectra were acquired with 4096 scans per point (ns) and 1.5 s mixing time.

The ^1^H–^15^N SOFAST-HMQC experiments were performed with 20 μM uniformly ^15^N-labeled IAPP in the absence or presence of 100 μM ligand, in 25 mM sodium phosphate buffer (pH 6.8) with 1% v/v DMSO-*d*_6_ at 5 °C. The ^15^N-labeled IAPP was produced recombinantly, complete with the disulfide bond and C-terminal amidation, as described previously.^30^ Spectra were acquired using a Bruker Avance III-HD 600 MHz spectrometer, with 128 scans per point (ns), relaxation delay (d1) of 0.3 s, and acquisition times of td1 = 40.4 ms and td2 = 106.9 ms.

Chemical shift assignments of human IAPP were reported previously.^26^ The spectra were recorded using Topspin 3.2 software (Bruker) and analyzed with CCPNMR 2.4.2 software.^64^ Residue-specific intensity ratios (*I*/*I*_0_) were calculated from the ^1^H–^15^N SOFAST-HMQC spectra, where I is the intensity of cross-peaks in the presence of the ligand and *I*_0_ is the intensity of cross-peaks of the protein alone. Chemical shift perturbations were calculated using the formula
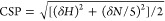
15

### Quantitation of Soluble and Fibril-Bound Compound

To determine the concentration of compound bound to fibrils at the end of self-assembly, 10 μM IAPP was incubated with 50 μM compound (YX-I-1, canagliflozin, doxazosin, or dapagliflozin) in 160 mM ammonium acetate (pH 7.4) with 1% v/v DMSO in a low-binding microplate (Corning 3881) at 30 °C, to allow fibril assembly to occur. After 24 h, the contents of the wells were aspirated with vigorous mixing to dislodge any fibrils adhering to the plate surface. The well contents were centrifuged for 30 min at 16,300 *g* (in a 200 μL volume) to pellet any fibrils and adhering compound, acquiring absorbance spectra (240–400 nm) before and after pelleting to observe the reduction in the compound peak. For data analysis, the compound peak was reference-subtracted (fibrils without compound, before and after pelleting) and baselined by subtracting any residual optical density at 400 nm to eliminate the confounding effects of absorption by IAPP or light scattering by fibrils. The proportion of each compound that had been copelleted was determined from the fold-change in absorbance at its λ_max_ (YX-I-1, 252 nm; canagliflozin, 291 nm; doxazosin, 340 nm; dapagliflozin, 276 nm).

To determine the concentration of compound that bound to preformed fibrils, 10 μM IAPP was used to prepare fibrils according to the same protocol described above, but without compound present (ie. DMSO vehicle only). The fibrils were extracted from the plate wells and incubated with 50 μM compound for 30 min at 30 °C in a low-binding microfuge tube (Eppendorf, Hamburg). The tube was then centrifuged for 30 min at 16,300 *g* (in a 200 μL volume) to pellet any fibrils and adhering compound, acquiring absorbance spectra (240–400 nm) before and after pelleting and quantifying the extent of copelleting in the same manner as described above.

For the Capflex experiments, 10 μM IAPP fibrils were prepared according to the same protocol as the absorbance-based copelleting experiments, with varying amounts of compound: (i) no compound (DMSO only); (ii) 20/30/40/60/100 μM canagliflozin, present during assembly; (iii) 100 μM canagliflozin, added after assembly; and (iv) 100 μM doxazosin, present during assembly. Compound-only controls that had been treated in the same manner were also prepared. Each preparation was placed in the autosampler of a Fida-1 instrument (FidaBio) in a glass pressure vial, with the autosampler and capillary both equilibrated to 30 °C. Capflex runs were performed as described in the “Capflex and TDA” section. To correct for differences in the intrinsic fluorescence of the compounds, Capflex elugrams were normalized relative to the plateau fluorescence of the relevant control containing a 100 μM compound. Spikes were identified using a threshold of 0.01 normalized fluorescence units above the running median fluorescence (window size 15). Capflex data were analyzed in the Fida analysis software supplied with the instrument and GraphPad Prism 10.

### Cryo-EM Data and Code Availability

Raw TIFF movies from cryo-EM have been deposited with EMPIAR and are publicly available as of the date of publication. Accession numbers are EMPIAR-12378 (IAPP-only control data set, with replicates EMPIAR-12383, EMPIAR-12384, and EMPIAR-12382), EMPIAR-12379 (IAPP + doxazosin data set), EMPIAR-12380 (IAPP + YX-I-1 data set), and EMPIAR-12381 (IAPP + canagliflozin data set). Final refined cryo-EM maps and models have been deposited in the EMD and PDB, respectively. Accession numbers for maps/models from the IAPP-only control are EMD-51730/PDB-9GZP (LL), EMD-51733/PDB-9GZS (LLU), EMD-51734/PDB-9GZT(LLUU), and EMD-51726/PDB-9GZ6 (2PF*S*). Accession numbers for maps/models from the IAPP + doxazosin data set are EMD-51735/PDB-9GZW (LL), EMD-51736/PDB-9GZX (LLU), and EMD-51737/PDB-9GZY(LLUU).

### Cryo-EM Sample Preparation and Data Collection

Monomeric IAPP was purified as described in “Pre-assay SEC”. Reactions were set up in duplicate in brown glass vials, with a total volume of 250 μL. Each reaction contained 30 μM IAPP with or without 50 μM compound (doxazosin, YX-I-1, or canagliflozin) in 160 mM ammonium acetate (pH 7.4) with 1% v/v DMSO. A further two replicates of the compound-free control were independently set up in a similar manner using a different preparation of monomeric IAPP, to help probe the reproducibility of the observed fibril polymorphism. Fibrillization progress was checked by removal of aliquots of reaction and measurement of the ThT fluorescence in a plate reader, where the final mixture contained 10 μM fibril reaction and 5 μM ThT in the same buffer as above. After quiescent incubation for 2 weeks at room temperature, at which point all reactions were at steady-state by ThT fluorescence, 4 μL of each sample was deposited onto 60 s plasma-cleaned (Tergeo) lacey carbon 300 mesh (Agar scientific) cryo-EM grids. Grids were prepared in a Vitrobot IV (ThermoFisher) at 6 °C and 90% humidity, with a wait time of 5 s and blot time of 6 s, and immediately plunge-frozen in liquid ethane. For each reaction, cryo-EM data sets of ∼2000 TIFF movies were collected on the Titan Krioses (ThermoFisher) at the Astbury Centre, University of Leeds as described in Table S9a–c.

### Cryo-EM Data Processing

Cryo-EM data sets were processed using a common framework, as outlined in Figure S14. Briefly, all movies were motion-corrected (RELION4^65^), with CTF estimation carried out on the resulting micrographs (CTFFIND4^66^). Filaments were manually picked on a subset of ∼100 micrographs and extracted into segments to train a picking model for autopicking all of the micrographs (crYOLO^67^). Picked segments were initially extracted 3× binned with a box size of ∼675 Å^2^ and cleaned using two rounds of 2D classification (all subsequent steps in RELION4 unless otherwise stated), with only nonfibrillar picking artifacts removed. The cleaned fibril segment data sets were then subjected to two sequential routes involving multiple classifications, first polymorph identification and then structure determination, as described below.

For polymorph identification, the aim was to identify all the resolvable fibril types present in the data, generating refined templates with distinct peptide backbone paths visible with optimized helical parameters (twist and rise). Due to the large numbers of particles/segments and complexity of twists evident in 2D classes of each data set, a key step was to use TypeCluster^23^ to separate filaments into groups by hierarchical clustering of segments. This generated 3–6 smaller groups containing related fibril types to simplify further processing/classification steps. In each cluster, 3D classification was initiated using initial templates generated from distinct 2D class averages and estimated helical crossovers from the data using relion_helix_inimodel2d.^68^ Multiple templates and helical twists were attempted for each cluster to identify as many unique fibril types as possible.

Structure determination was performed after polymorph identification, going back to the complete cleaned fibril segment data set with the now identified and refined polymorph templates (and corresponding optimized helical twists). Separate 3D classification runs were performed with each different template in a sequential manner, so that all particles were classified with polymorph 1, corresponding output classes were saved, remaining classes were combined and classified with polymorph 2, and so on. All polymorph distributions (Figure 8a–c) were calculated from these 3D classification runs based on appearance/presence of complete backbone paths in output class averages, with ambiguous/unfeatured classes (after trying with every identified polymorph as template) combining to give the “unresolved” group of segments. After this point, the best-resolved classes were selected for a high-resolution structure solution for each polymorph. Final resolved structures for the control and doxazosin-grown data sets are shown in Figure S16a–d, with gold-standard resolutions calculated at 0.143 FSC (Figure S16e,f) and full data statistics reported in Tables S10 and S11.

A 3D structure of the canagliflozin-grown IAPP fibrils could not be obtained due to the lack of detectable helical twisting or easily identifiable boundaries between protofilaments within the fibril-containing sheets. Efforts to align the segments and detect subtle twists or twisting subpopulations were not successful, including the use of TypeCluster^23^ with 2D classification. Attempts at 3D classification and refinement with either no twist or a very small twist (0.1 ^o^/layer) also failed to yield any reliable structural features. Finally, electron diffraction was attempted, but the sheet-like assemblies were too thin to withstand the beam and generate diffraction.

### Cryo-EM Model Building and Validation

For the 2PF^S^ structure in the IAPP-only control, PDB: 6ZRF(46) was docked into the map using ChimeraX^69^ and the first polypeptide chain was adjusted to fit the density using real-space refinement in Coot,^70^ while correcting any rotamer and Ramachandran plot outliers. The chain was then duplicated and rigid body fit into the map to create six layers of the two-subunit core. The model was globally real-space refined using Phenix,^71^ with NCS restraints to limit divergence of the repeating layers, and then validated using MolProbity.^72^ For the 6 remaining structures (LL/LLU/LLUU in the IAPP-only control and doxazosin-grown data sets), a similar process was performed, starting with de novo built L- and U-fold subunits for the first instance and then docking built models as templates for subsequent structures. Each structure was manually adjusted where clear density differences were present and independently real-space refined in Phenix. The final refinement and model statistics for all the deposited structures are reported in Tables S10 and S11.

### Filament Crossover Measurements

Filament crossover measurements (Figure 7b) were made from ∼100 randomly selected fibril-containing cryo-EM micrographs for each sample. In each case, the main cryo-EM data set processed for structure analysis was used, as well as one data set from a replicate reaction to ensure that the patterns observed were sample-specific. For measurements, images were opened in Fiji,^73^ the correct pixel scale was set, and crossovers were measured on all distinct fibrils that could be classified. In each case, some fibrils were excluded where classification was ambiguous, typically when there was a variable crossover or a high degree of overlap with other fibrils. For the canagliflozin-grown samples, sheets were purposefully ignored from the count that led to the final plot, as their large and varied width meant that the amount of IAPP they contained could not be quantified in a comparable manner.

### Seeded IAPP Self-Assembly Reactions

Seeds were prepared by incubating the SEC-purified IAPP monomer under the same conditions used for kinetic assays (160 mM ammonium acetate pH 7.4, 1% v/v DMSO, low-binding 96-well plates at 30 °C) in the absence or presence of 50 μM doxazosin, YX-I-1, or canagliflozin. After 24 h, the contents of reactions were extracted from plate wells, pooled in volumes of 250 μL, and centrifuged for 30 min at 16,300 *g* to pellet aggregates. The supernatant was discarded, and the pellet was snap-frozen and stored at −20 °C. To start a seeded self-assembly reaction, the pellet was thawed, resuspended in 250 μL of the same buffer (without a small molecule), and sonicated for 10 min. Resuspended seeds were added in a 30% ratio (3 μM) to assembly reactions containing 10 μM IAPP in 160 mM ammonium acetate (pH 7.4) with 1% v/v DMSO and 20 μM ThT, but no compound. Self-assembly was monitored by ThT fluorescence in low-binding 96-well plates in a CLARIOstar plate reader (BMG Labtech, UK) at 30 °C, using the same protocol used for other kinetic assays. Two biological repeats were performed with three replicate wells (100 μL each) per concentration and repeat. A high degree of concordance was observed between repeats.

## Data Availability

All data will be available on publication in Leeds DOI (10.5518/1604).

## Supporting Information Available

Preliminary SAR supporting the importance of the tetrahydropyran-containing portion of YX-I-1 for its activity, **s**ummary of FIDA techniques, small molecule solubility screening, IAPP self-assembly kinetics under the screening conditions, further analysis of inhibitor screening data, further controls to support the kinetic screening and analysis, SPR titration of canagliflozin with IAPP, protein-detected NMR of IAPP with small molecules, further cryo-EM images, analysis, and explanation of processing workflow, effect of small molecules on the seeding potency of IAPP amyloid, summaries of the virtual screening data and compounds identified, summary of small molecule solubility screening, analysis of IAPP self-assembly kinetics under the screening conditions, summary of inhibitor screening data, analysis of IAPP self-assembly kinetics with varying inhibitor concentrations, and cryo-EM data collection and refinement statistics (PDF)
