# Supplementary material for: Kinetic Steering of Amyloid Formation and Polymorphism by Canagliflozin, a Type-2 Diabetes Drug
Source: J Am Chem Soc. 2025 Feb 21;147(14):11859–78. doi: 10.1021/jacs.4c16743 (PMC11987024; doi:10.1021/jacs.4c16743)
Supplement: Supplementary file 1 — ja4c16743_si_001.pdf [file ja4c16743_si_001.pdf]

Supporting Information

Kinetic Steering of Amyloid Formation and Polymorphism by Canagliflozin, a Type-2 Diabetes Drug

Alexander I. P. Taylor[1,2]*, Yong Xu[1,2], Martin Wilkinson[1], Pijush Chakraborty[1], Alice Brinkworth[1], Leon F. Willis[1], Anastasia Zhuravleva[1], Neil A. Ranson[1], Richard Foster[2], and Sheena E. Radford[1]*

[1]Astbury Centre for Structural Molecular Biology, School of Molecular and Cellular Biology, Faculty of Biological Sciences, University of Leeds, LS2 9JT, UK

[2]Astbury Centre for Structural Molecular Biology, School of Chemistry, Faculty of Engineering and Physical Sciences, University of Leeds, LS2 9JT, UK

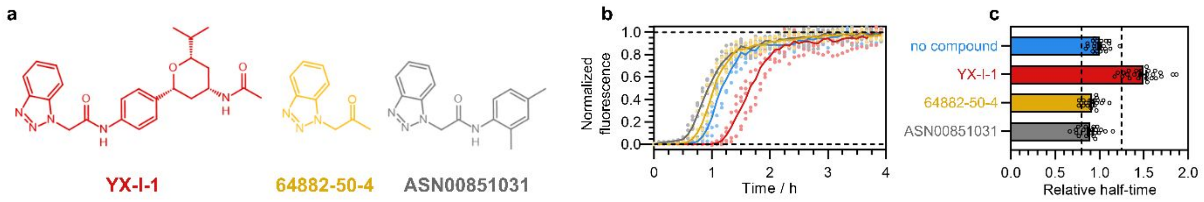

Figure S1: Preliminary structure-activity relationship (SAR) suggests that the tetrahydropyran-containing portion of YX-I-1 is important for its activity. a Two readily available YX-I-1 analogues, 64882-50-4 and ASN00851031, which lack the tetrahydropyran ring and its substituents, were tested for inhibition of IAPP amyloid formation using ThT assays, with 4 biological repeats per analogue (5 for YX-I-1) and 5 replicate wells per repeat. Conditions were the same as used in the later screen (160 mM ammonium acetate, pH 7.4, 1% v/v DMSO, 10 μM IAPP, 50 μM compound, low-binding plates at 30°C; see **Methods**). **b** Example of the effects of YX-I-1, 64882-50-4, and ASN00851031 on IAPP aggregation kinetics, from the first biological repeat. Blue (no compound), red (YX-I-1), yellow (64882-50-4), black (ASN00851031). **c** Summary of the fold-change in half-time of amyloid formation across all repeats. Each dot represents a single replicate well, and error bars show the standard error of the mean across all replicates. The dashed lines represent fold changes of 0.8x and 1.25x used as thresholds for determining activity.

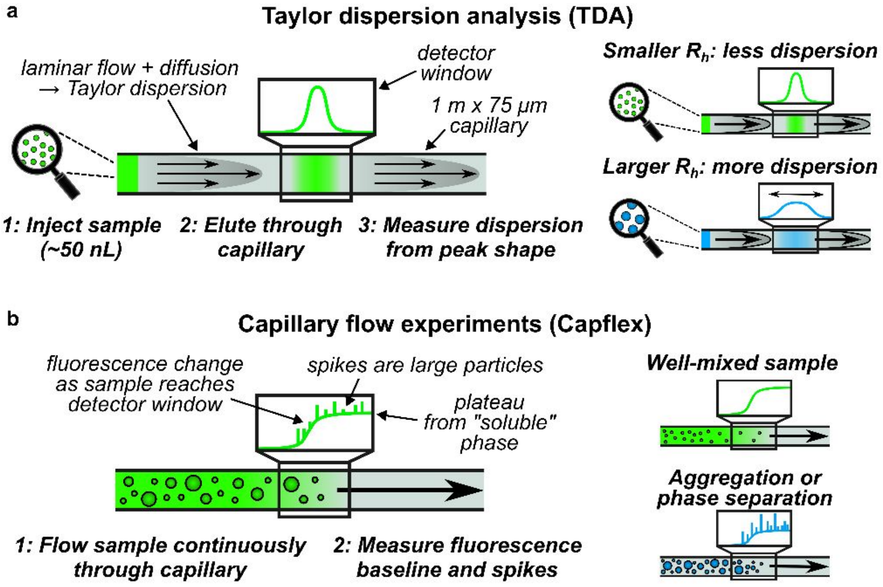

Figure S2: Summary of FIDA techniques to study molecular solubility, conformation and aggregation. a Schematic of Taylor dispersion analysis (TDA). A ~50 nL plug of sample is introduced into a pre-equilibrated capillary and eluted with the same buffer. A combination of laminar flow and diffusion orthogonal to the direction of flow causes Taylor dispersion[1], in which the plug spreads out at a rate proportional to the Stokes radius (R_h). Measuring the dispersion (by intrinsic or extrinsic fluorescence) with known capillary dimensions and flow parameters allows determination of the R_h, with a typical accuracy of 5% and range of 0.25-500 nm (**Methods**). **b** Schematic of Capflex[2], performed on the same instrument. Capflex takes advantage of the Fida-1 instrument's ability to resolve large particles (approx. >1 µm) as discrete fluorescence spikes. Unlike TDA, sample is flowed continuously into the capillary, providing better spike-counting statistics. The "soluble" phase, i.e. particles that are truly dissolved, or otherwise small and homogeneously distributed, contributes to the sigmoidal increase in fluorescence baseline as the sample reaches the detector window, which can be measured quantitatively. Phase separation of part of the sample into condensates or solid particles will cause a reduction in the fluorescence intensity at steady state, and the appearance of discrete spikes[2].

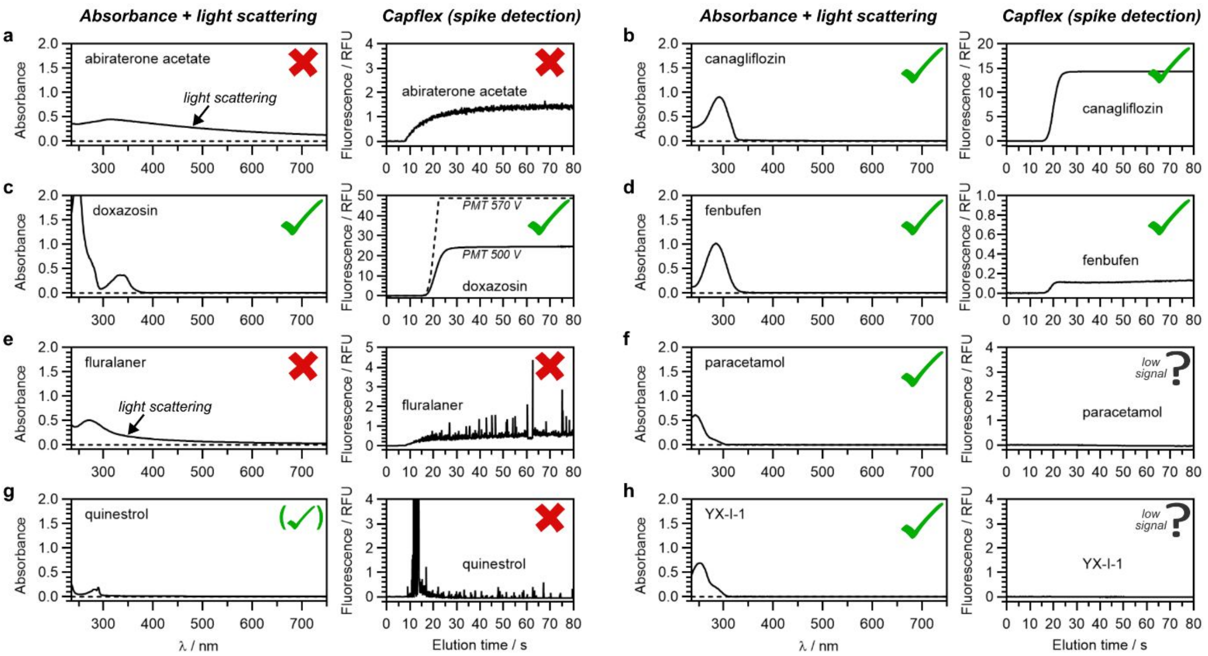

Figure S3: Small molecule solubility screening by absorbance spectroscopy and Capflex. Panels **a-h** show examples of absorbance spectra from 235-750 nm (left) and Capflex elugrams (right) of small molecules under the screening conditions (160 mM ammonium acetate pH 7.4, 1% v/v DMSO, 50 µM compound). Small molecules shown in this figure were selected to represent a range of screening outcomes, as well as including key molecules dealt with in this study. In the absorbance spectra, light scattering caused by sufficient concentrations of insoluble particles can be detected as a sloping, non-zero baseline (eg. at 600 nm) (**a, e**), and was confirmed by collecting further spectra at a range of compound concentrations and after centrifugation (**Methods**). In Capflex elugrams, insoluble particles can be detected directly as spikes with high intrinsic fluorescence, even at very low concentrations (**a, e, g**). However, the solubility of certain compounds could not be assessed by Capflex, due to their low intrinsic fluorescence (**f, h**). Compounds that had no evidence of insoluble particles by either technique (**b-d**) were passed. Note that doxazosin (**c**) had to be re-run with reduced detector sensitivity (PMT voltage of 500 V vs. 570 V), due to its very high intrinsic fluorescence. For more details of solubility screening using both techniques, see **Methods**.

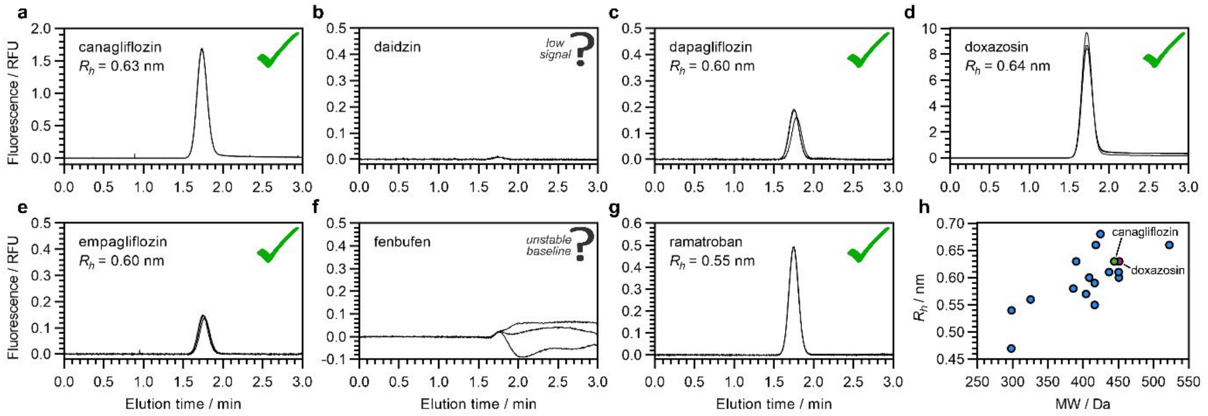

Figure S4: Small molecule R_h determination using Taylor dispersion analysis (TDA). As an additional quality control, compounds that passed solubility screening and were sufficiently fluorescent (**Fig. S3**) were analyzed by TDA (**Fig. S2a**) to determine their in-solution R_h. Panels **a-g** show example elugrams for 7/19 compounds assayed using TDA. In most cases (17/19; panels **a, c, d, e, g**), the R_h could be determined, but daidzin (**b**) had insufficient signal-to-noise, and fenbufen (**f**) was affected by an unstable baseline. The range of R_h values (0.47-0.68 nm (**Table S5**)) is as expected for the small molecules (298.3-522.6 Da). As shown in **h**, compound R_h is correlated with molecular weight (Pearson r = 0.7509). Canagliflozin (green) and doxazosin (purple) are highlighted, other compounds are shown in blue.

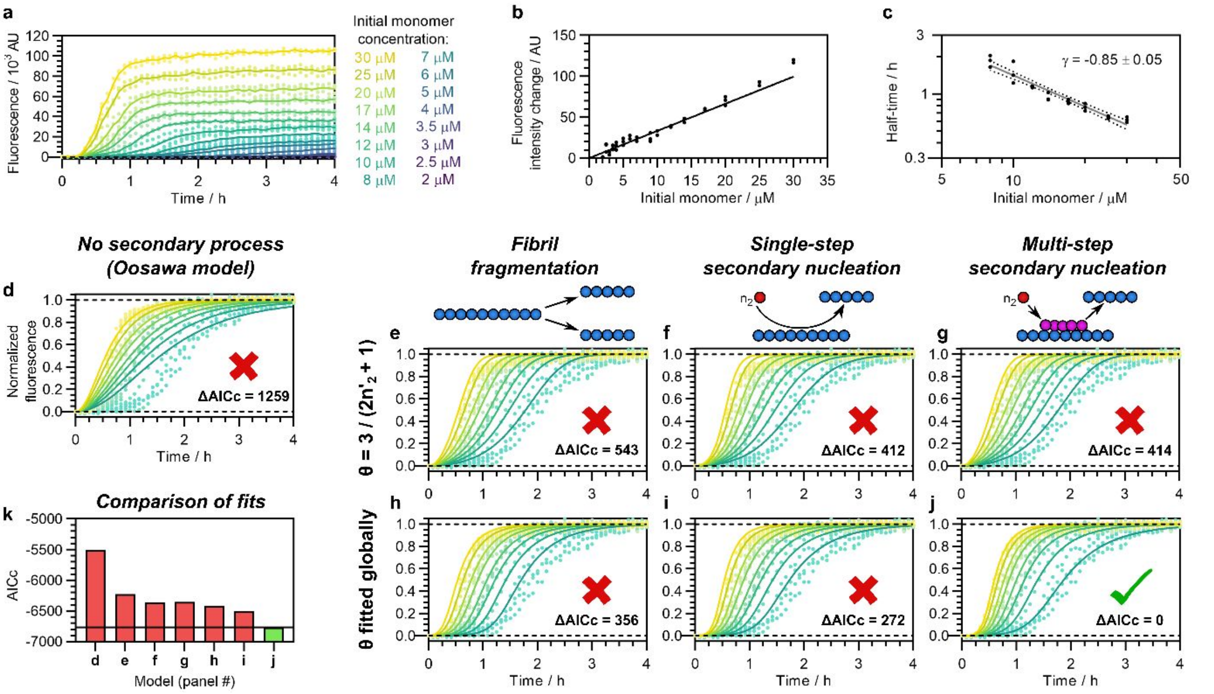

Figure S5: IAPP self-assembly under the screening conditions follows a multi-step secondary nucleation-dominated mechanism. a Blank-subtracted ThT fluorescence curves showing IAPP amyloid formation at 2-30 μM IAPP (160 mM ammonium acetate pH 7.4, 1% v/v DMSO, low-binding 96-well microplates, 30°C, quiescent). **b** ThT fluorescence intensity change from the start to the end of self-assembly, showing a linear relationship between fibril yield and ThT fluorescence intensity. **c** Double-logarithmic plot of the amyloid self-assembly half-time of 8-30 μM IAPP. Kinetics at lower IAPP concentrations were not analyzed, as half-times were unreliable and the curve shape often biphasic; implying that an IAPP concentration of at least 8 μM is needed for a single self-assembly pathway to dominate. Fitting the half-times for 8-30 μM IAPP to the power law $\tau_{50} \propto m(0)^{\gamma}$ yielded a scaling exponent of $\gamma = -0.85$, close to $\gamma = -0.82$ previously reported for 8-32 μM IAPP[3]. **d-k** Model fitting and comparison, using the normalized ThT fluorescence curves at 8-30 μM IAPP. Oosawa's exact solution (**d**) fails due to its lack of secondary processes. For models with secondary processes, fits with the internal parameter θ, which controls the shape of the late growth phase, were set as either $\theta = 3/(2\,n_2' + 1)$ (**e-g**) [4], or fitted empirically while shared across concentrations (**h-j**). We considered three dominant secondary processes: (**e, h**) fragmentation, (**f, i**) single-step secondary nucleation, and (**g, j**) multi-step secondary nucleation (**Methods**). While theories often link θ to the order of the dominant secondary process[4,5], this assumes that identical processes predominate in the lag phase and late growth phase; however, appearance of late-stage processes (eg. flocculation, fibril maturation) will break this link. Here, the failure of **e-g**, caused by a mismatch between late-growth-phase curve shape and global concentration dependence, shows unequivocally that additional uncharacterized processes must occur in the late growth phase. Out of **h-j**, multi-step secondary nucleation (**j**) is the clear best fit, as quantified

by Akaike's corrected information criterion (AICc) (**k**), and the corresponding ΔAICc values (**d-j**), defined as the difference in AICc between the fit in question and the best fit (**j**). The fitting comparison agrees with previous findings that IAPP has multi-step secondary nucleation as a dominant secondary process[3]. Full details of modelling are in (**Methods**), and fitted parameters are in **Table S6**.

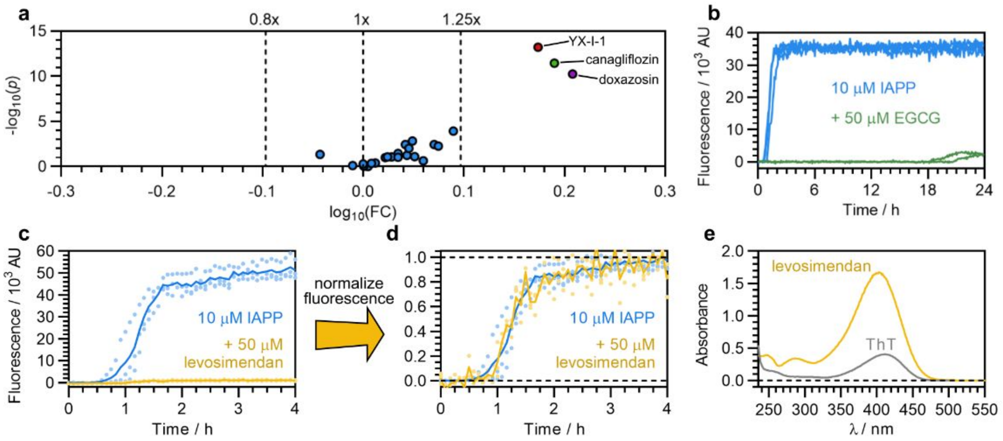

Figure S6: Further analysis of small molecule activity screening. a Volcano plot showing the statistical significance (using *p*-values from Mann-Whitney *U* tests) versus log-fold-change in IAPP self-assembly half-time in the presence of molecules in the activity screening set, against the DMSO-only control (from **Table S7**). The equivalent *p*-value for YX-I-1 has also been included for comparison. Color scheme: red, YX-I-1; green, canagliflozin; purple, doxazosin; blue, other compounds. Dashed lines represent the magnitude threshold used to establish activity (**Methods**). **b** EGCG, used as a positive control, caused a pronounced increase in IAPP self-assembly half-time. **c-d** In the screen, levosimendan produced a marked reduction in ThT fluorescence intensity. However, the normalized ThT kinetics showed no change in the half-time (see **Fig. 2b, Table S7** for summary). **e** There is a close overlap between the absorbance spectra of levosimendan (50 μM) and ThT (20 μM); thus, attenuation at the wavelengths used to excite ThT is the probable cause of reduced ThT fluorescence.

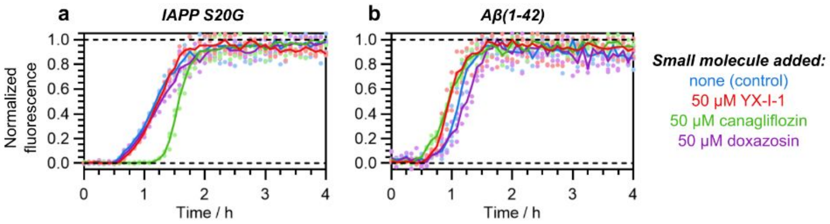

Figure S7: Kinetics of IAPP S20G and Aβ(1-42) self-assembly in the presence of YX-I-1, canagliflozin, or doxazosin. Panels show the normalized self-assembly kinetics of (**a**) 10 µM IAPP S20G or (**b**) 4 µM Aβ(1-42) in the absence or presence of 50 µM YX-I-1, canagliflozin, or doxazosin, under the same conditions used for wild-type IAPP self-assembly experiments (160 mM ammonium acetate, pH 7.4, 1% v/v DMSO, 30°C, quiescent). The color scheme corresponds to the small molecule added: blue, none; red, YX-I-1; green, canagliflozin; purple, doxazosin. The solid line shows the running average over replicate wells.

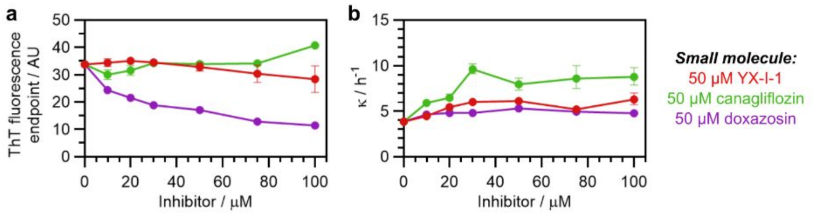

Figure S8: Further analysis of the dose-dependent effects of YX-I-1, canagliflozin and doxazosin on IAPP self-assembly kinetics. a Dependence of the ThT fluorescence intensity endpoint on the compound concentration, with error bars showing the standard error of the mean (n=3). **b** Extracted rate of the secondary pathway of IAPP fibril formation, κ, with error bars representing the fitting errors. The same data are plotted in **Fig. 3g** on a logarithmic scale to allow side-by-side comparison with the changes in λ, but are shown here on a linear scale to highlight the small changes in κ observed with canagliflozin. The color scheme for both panels corresponds to the small molecule added, as shown in the key on the right.

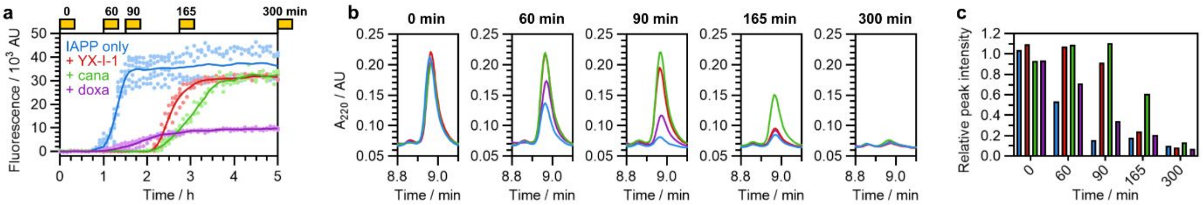

Figure S9: Fibril pelleting combined with monomer quantitation by analytical HPLC confirms that the inhibitors delay the onset of monomer loss. a A ThT assay was set up under standard conditions (160 mM ammonium acetate, pH 7.4, 1% v/v DMSO, 30°C, quiescent) with no compound, or 50 μM YX-I-1, canagliflozin, or doxazosin. At time points of 0/60/90/165/300 min, the reaction was paused and several replicate wells with each compound (or none) were extracted to quantify the remaining non-pelletable IAPP. Yellow flags mark the time at which the assay was paused, and the subsequent 20 min required for extraction of well contents and pelleting, during which aggregation will have continued. **b** Fibrils were pelleted by centrifugation for 15 min at 16,300 *g*, and the remaining IAPP in the supernatant was quantified by analytical HPLC. **c** Change in relative monomer peak intensity across successive sampling time points, calculated as the integral of the monomer peak (8.8-9.2 min) and normalized relative to the mean peak intensity of the initial time point. For all panels, the color scheme denotes the compound present: blue, no compound; red, YX-I-1; green, canagliflozin; purple, doxazosin. For full experimental details, see **Methods**.

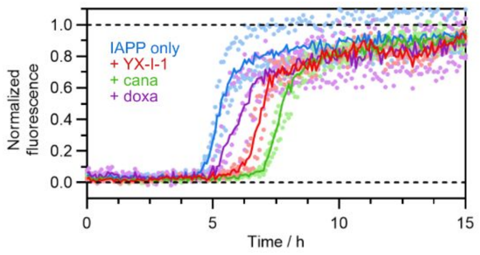

Figure S10: YX-I-1, canagliflozin, and doxazosin inhibit IAPP self-assembly in the buffer used for NMR experiments. Data show the normalized ThT fluorescence intensities for IAPP fibril assembly in 25 mM sodium phosphate (pH 6.8) with 1% v/v DMSO, at 30°C. Experiments were conducted without small molecules (blue), or with 50 µM YX-I-1 (red), canagliflozin (green), or doxazosin (purple). The solid line shows the running average over replicate wells.

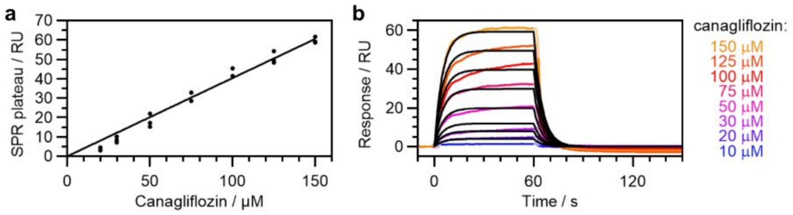

Figure S11: SPR titration of canagliflozin with IAPP. a The plateau of SPR experiments with varying concentrations of canagliflozin is linearly proportional to the concentration of canagliflozin over the entire range studied, indicating that the K_d is well above the highest concentration tested (150 µM). **b** Global fitting of the canagliflozin titration gave a good fit (R^2 = 0.99), but the K_d diverged towards arbitrarily high values (>> 150 µM), rather than converging. This implies that the K_d is well above the experimental range (and solubility limit) under these conditions, and cannot be determined by global fitting. The SPR titration was performed with varying concentrations of canagliflozin in 160 mM ammonium acetate (pH 7.4) with 1% v/v DMSO, at 30°C.

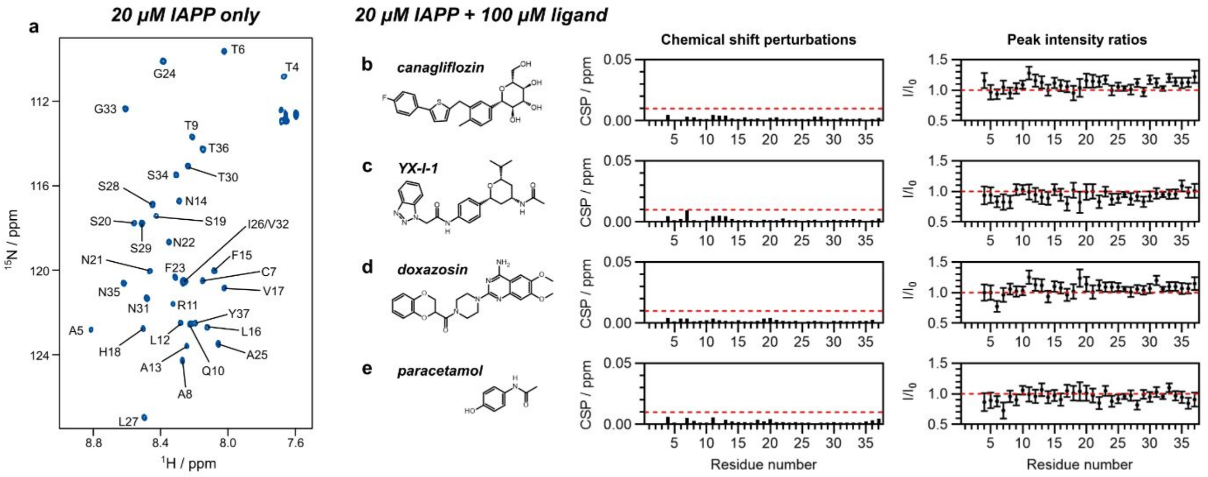

Figure S12: Protein-detected NMR of IAPP with or without small molecules. a ^{1}H-^{15}N SOFAST HMQC spectrum of 20 µM wild-type IAPP in 25 mM sodium phosphate (pH 6.8) with 1% v/v DMSO. **b-e** Effects of 100 µM small molecule on the spectrum of wild-type IAPP in the same buffer (left, compound and structure; center, chemical shift perturbations (CSPs); right, effect on peak intensity). The dashed red line in the plots of CSPs marks the threshold of 0.01 ppm used to determine whether significant CSPs were present. Error bars in peak intensity are calculated from the signal-to-noise ratio. See **Methods**.

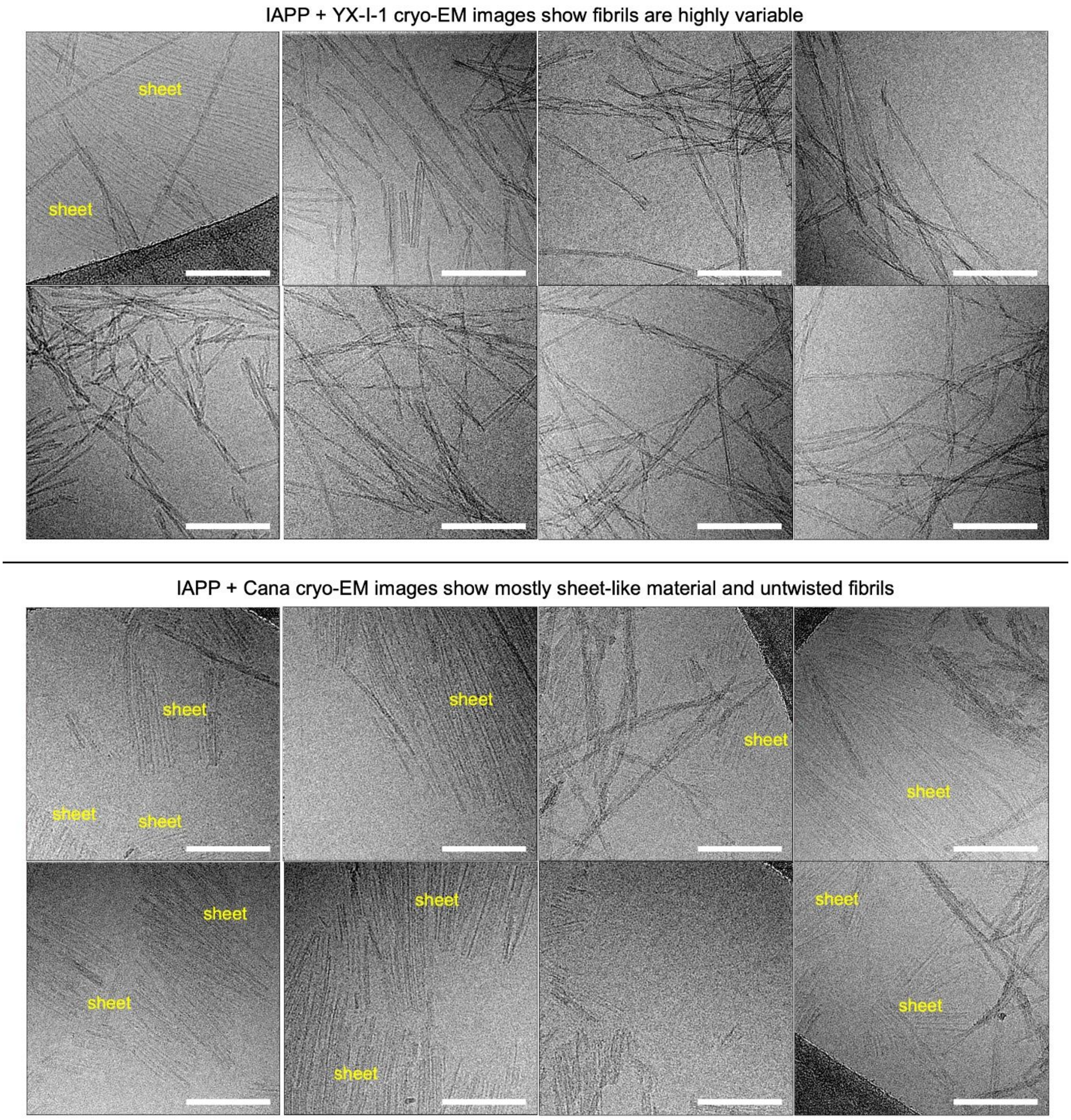

Figure S13. Cryo-EM images exemplifying the morphology of IAPP fibrils grown in the presence of YX-1-1 and canagliflozin. Representative micrographs are shown from the IAPP + YX-I-1 (*top*) and IAPP + canagliflozin (*bottom*) cryo-EM datasets. Sheet-like fibrillar material is labelled in the images, with occasional occurrence in the YX-I-1 dataset and high frequency (>90% of images) in the canagliflozin dataset. Scale bar represents 100 nm.

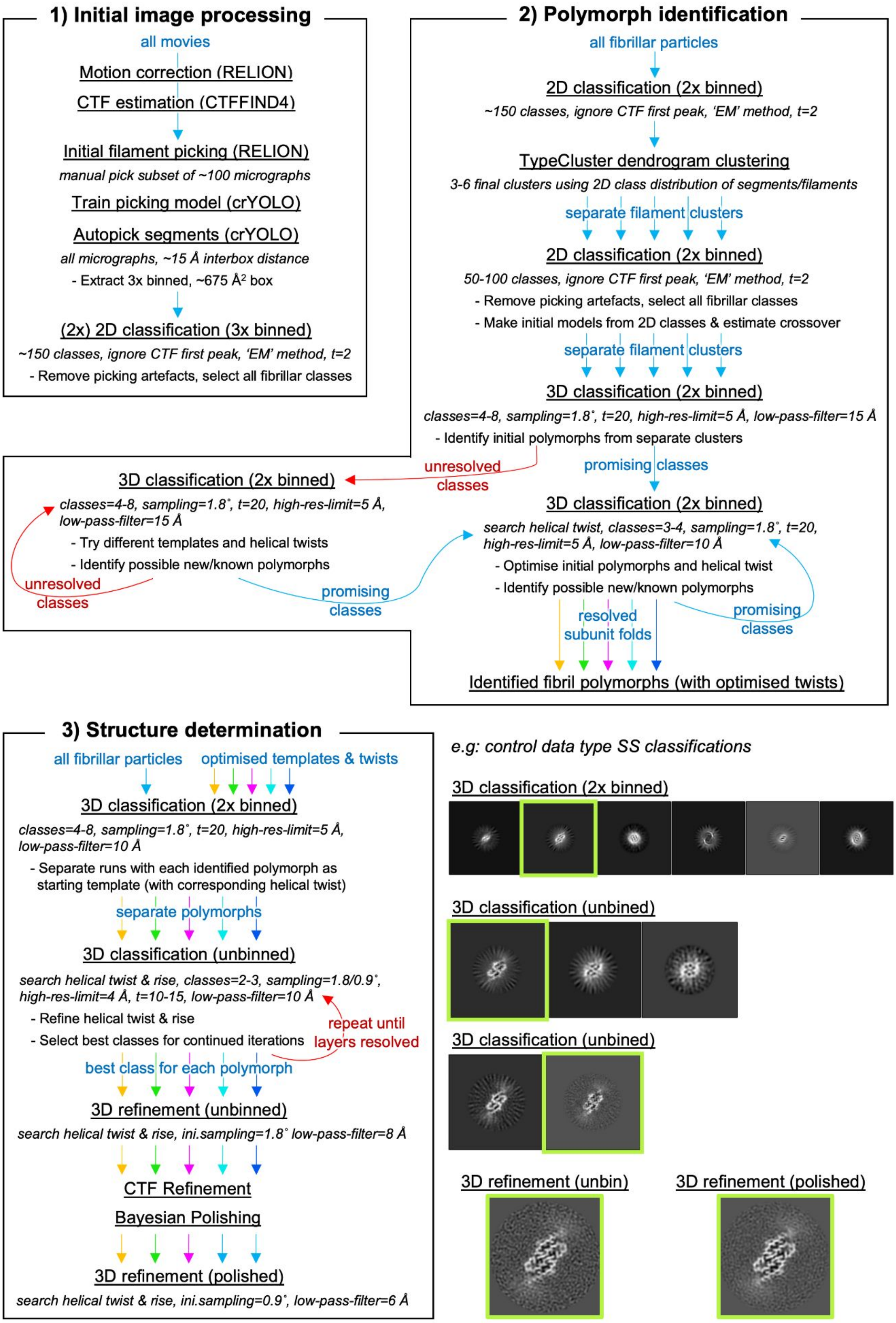

Figure S14. Detailed cryo-EM processing strategy to identify and resolve multiple fibril polymorphs within each dataset. The workflow was split into three stages, as described in the

Methods: 1) initial “image processing”, identical in all datasets, to generate a stack of all fibrillar particles; 2) “polymorph identification”, which involved multiple rounds and iterations of different classification approaches to identify different fibril types present; and 3) “structure determination”, in which the identified fibril types were used to completely classify all the fibrillar particles before iteratively classifying each group to reveal high-resolution structures from homogeneous sub-populations. In each stage, specific run parameters are described in italics. Example 3D classification outputs (*bottom right*) are shown for stage 3) with the IAPP $2PF^S$ fibril template and resulting path toward the final structure. Selected classes to progress to the proceeding run are boxed in lime green.

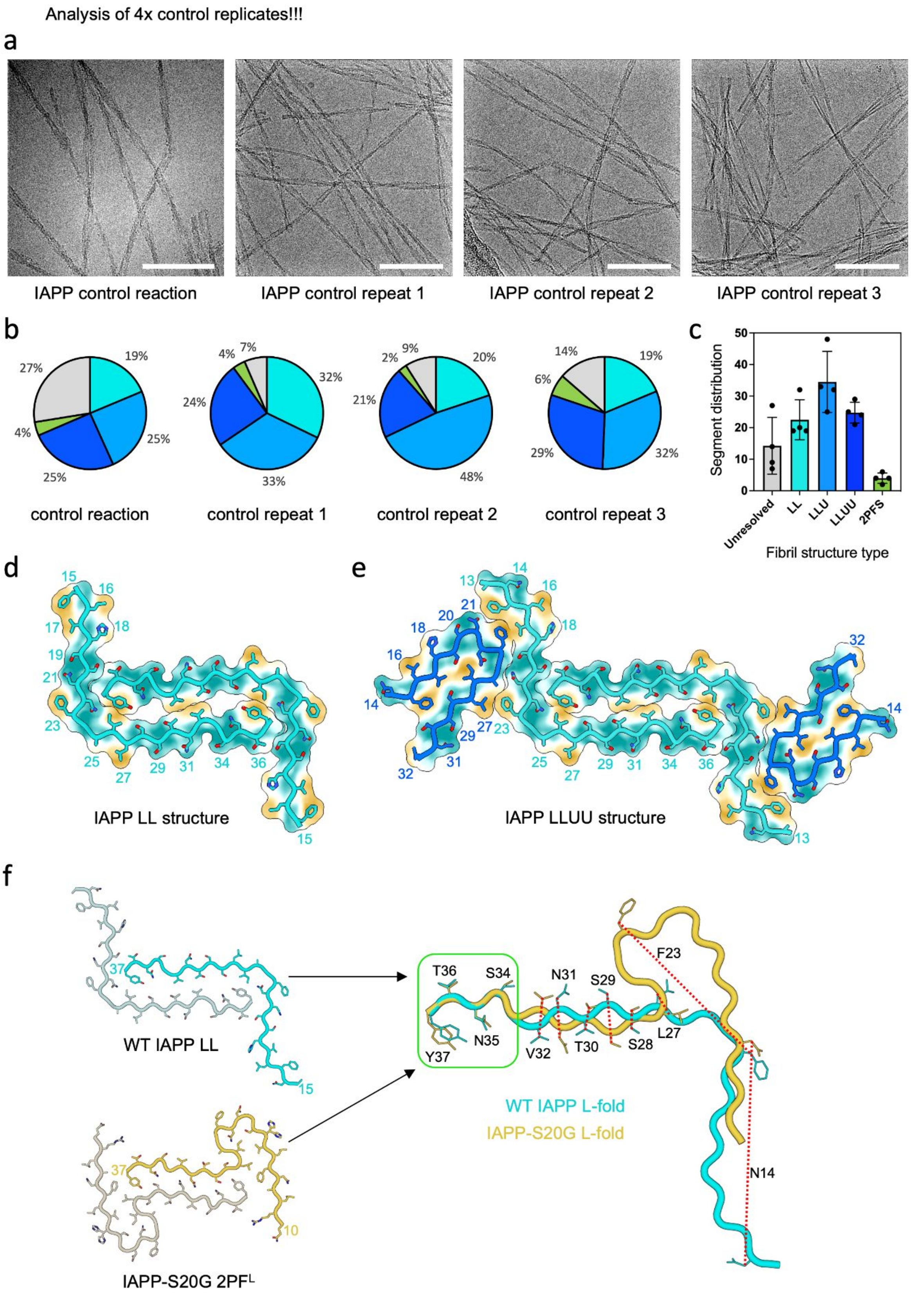

Figure S15. The polymorph distributions of IAPP-only control fibrils are reproducible across four independent reactions. a Representative micrographs for each dataset from the four distinct

IAPP-only control reactions, with scale bar of 100 nm. **b** Pie charts representing the distribution of fibril types after full classification of each dataset using the approach outlined in **Figure S14**. **c** Bar chart summarizing the distribution of fibril types, with each dataset shown as dots, the average as colored bars, and the standard deviation as error bars. **d-e** Final models of the LL and LLUU structures from the IAPP-only control, with the mainchain and sidechains colored according to subunit identity, and the surface representation superimposed and colored by the molecular lipophilicity potential of each atom (cyan, hydrophilic; gold, hydrophobic), as calculated in ChimeraX[6]. **f** The wild-type L-fold differs from the previously published IAPP-S20G $2PF^{L}$-fold (PDB: 8AZ4)[7]. A layer of each structure is shown as a ribbon diagram with sidechain sticks (*left*), next to a superposition of the two subunit folds aligned on the C-terminal five residues (*right*). Residues in the C-terminal strand and two intermediate positions along the chain are marked with dashed red lines between matching C_{α} atoms to indicate structural deviation. Only the C-terminal five residues are superimposed in this alignment (green box).

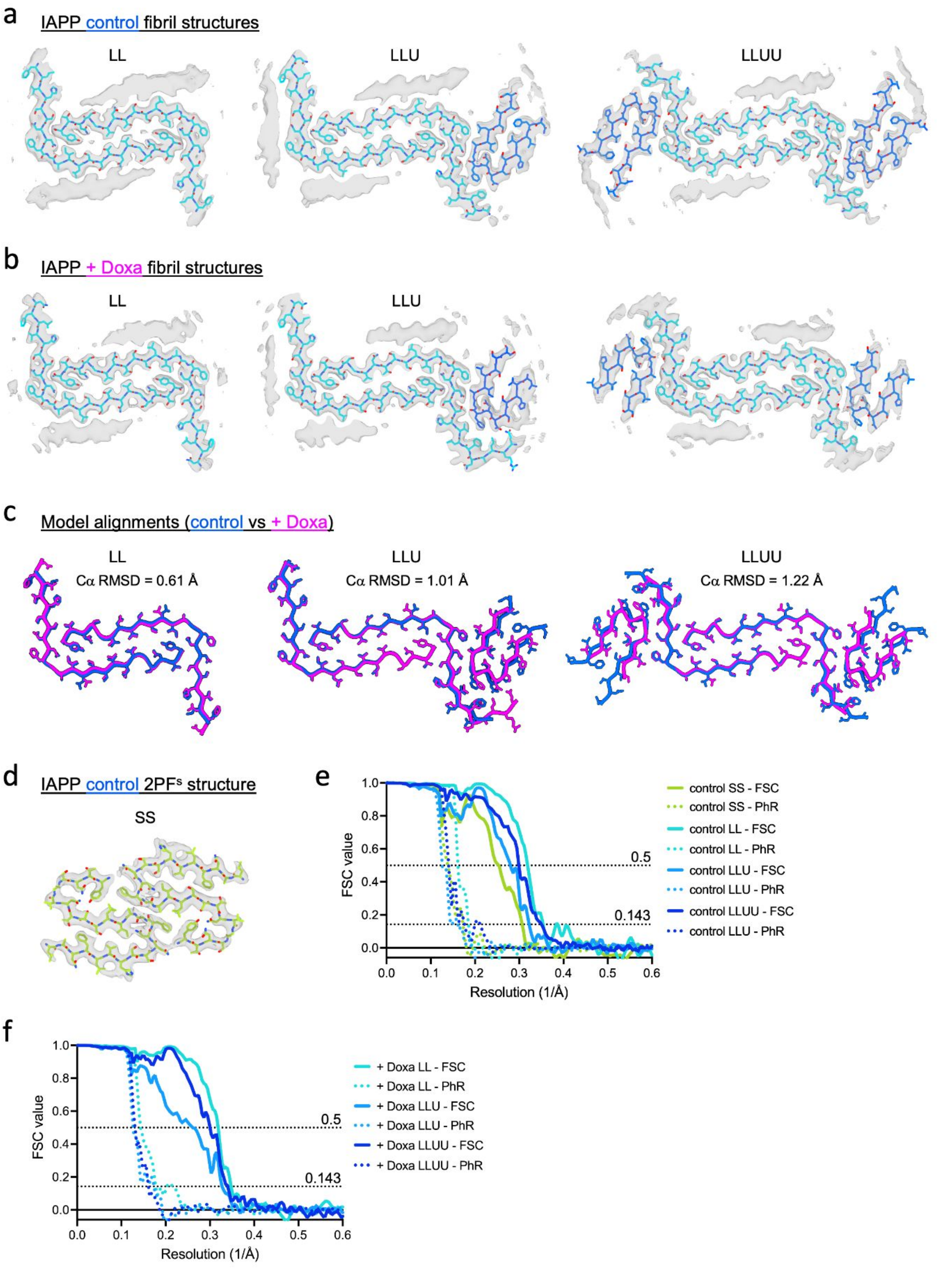

Figure S16. Comparison of the final structures of IAPP fibrils from the compound-free control and doxazosin-containing datasets. a The deposited models (shown as sticks colored by subunit fold) and maps (transparent gray surfaces) for the LL, LLU, and LLUU fibril structures in the IAPP-only control dataset. **b** The same, but for the doxazosin-containing dataset. In both cases, additional poorly

resolved β-strands were present in the density that could not be modelled. **c** Superpositions of one layer of the control (blue) and doxazosin-grown (magenta) structures for each fibril type, alongside the mean C_{α} RMSD values calculated across all residues. **d** The deposited model and map for the control $2PF^{S}$ fibril structure. **e-f** FSC plots from the corresponding RELION postprocessing run for each deposited structure from the control and doxazosin-grown datasets, with the corrected final FSC curves shown as solid lines, and the phase-randomized (PhR) FSC curves shown as dashed lines.

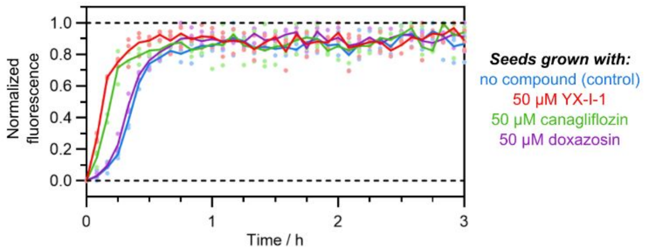

Fig. S17: Effect of small molecules on the seeding potency of IAPP amyloid. Seeds were prepared by incubating 10 µM IAPP monomer for 24 h in 160 mM ammonium acetate (pH 7.4) with 1% v/v DMSO at 30°C, in the absence or presence of 50 µM YX-I-1, canagliflozin, or doxazosin. Seeds were then extracted by centrifugation and used to initiate new compound-free IAPP self-assembly reactions with a 30% seeding ratio (3 µM seed to 10 µM monomer) (**Methods**). Data show the normalized ThT fluorescence intensity of IAPP monomer in the presence of seeds grown with or without compound. The color scheme on the right of the figure represents the compound (or none) present during preparation of the seed (but *not* the seeded self-assembly reaction itself).

Table S1: Tetrahydropyran-containing compounds identified by substructure searches, with ROCS data for comparison.

Name	CAS identifier	Compound class	Tetrahydropyran-containing moiety	ROCS combo score (#rank)		Notes
				Enantiomer 1	Enantiomer 2	
canagliflozin	842133-18-0	flozin	*C*-glucosyl	0.759 (#45)	0.577 (#453)	
daidzin	552-66-9	isoflavone glucoside	*O*-glucosyl	0.787 (#22)	0.595 (#307)	
dapagliflozin	461432-26-8	flozin	*C*-glucosyl	0.688 (#246)	0.634 (#111)	
empagliflozin	864070-44-0	flozin	*C*-glucosyl	0.613 (#816)	0.610 (#218)	
ertugliflozin	1210344-57-2	flozin	*C*-glucosyl	0.628 (#678)	0.564 (#555)	
ipragliflozin	951382-34-6	flozin	*C*-glucosyl	0.664 (#407)	0.534 (#888)	
licogliflozin	1291094-73-9	flozin	*C*-glucosyl	N/A	N/A	
liquiritin	551-15-5	flavanone glucoside	*O*-glucosyl	0.737 (#75)	0.609 (#220)	
polydatin (piceid)	65914-17-2	stilbenoid glucoside	*O*-glucosyl	0.728 (#98)	0.621 (#160)	
remogliflozin A	329045-45-6	flozin	*O*-glucosyl	N/A	N/A	active form
remogliflozin etabonate	442201-24-3	flozin	*O*-glucosyl	N/A	N/A	prodrug
sotagliflozin	1018899-04-1	flozin	*C*-glucosyl	0.650 (#510)	0.653 (#63)	
tofogliflozin	1201913-82-7	flozin	*C*-glucosyl	0.637 (#596)	0.600 (#263)	
velagliflozin	946525-65-1	flozin	*C*-glucosyl	N/A	N/A	

**Table S2: Ranked ROCS hits for YX-I-1 enantiomer 1, from searching the FDA-approved drug library.**						
**Rank**	**Name**	**CAS identifier**	**Tanimoto similarity**			**Notes**
			**Shape**	**Color**	**Combo**	
Compounds in the top 10 by combo score.						
1	tivozanib	475108-18-0	0.656	0.258	0.914	Selected for further investigation.
2	doxazosin	74191-85-8	0.625	0.239	0.864	Selected for further investigation.
3	phthalylsulfathiazole (PSTZ)	85-73-4	0.677	0.178	0.855	Selected for further investigation.
4	ramatroban	116649-85-5	0.652	0.195	0.847	Selected for further investigation.
5	fenbufen	36330-85-5	0.546	0.289	0.835	Selected for further investigation.
6	benzonatate	32760-16-0	0.537	0.280	0.817	*Excluded from screen (sold with PEGylation of varying length).*
7	fluralaner	864731-61-3	0.461	0.355	0.817	Selected for further investigation.
8	rafoxanide	22662-39-1	0.671	0.144	0.816	Selected for further investigation.
9	fursultiamine	804-30-8	0.535	0.278	0.813	*Excluded from screen (potentially labile disulfide bond).*
10	piribedil	3605-01-4	0.567	0.243	0.810	Selected for further investigation.

**Table S3: Ranked ROCS hits for YX-I-1 enantiomer 2, from searching the FDA-approved drug library.**						
**Rank**	**Name**	**CAS identifier**	**Tanimoto similarity**			**Notes**
			**Shape**	**Color**	**Combo**	
Compounds in the top 10 by combo score.						
1	fenbufen	36330-85-5	0.498	0.295	0.793	Selected for further investigation.
2	abiraterone acetate	154229-18-2	0.470	0.253	0.723	Selected for further investigation.
3	levosimendan	141505-33-1	0.470	0.250	0.720	Selected for further investigation.
4	piribedil	3605-01-4	0.501	0.215	0.716	Selected for further investigation.
5	tivozanib	475108-18-0	0.466	0.246	0.713	Selected for further investigation.
6	pexidartinib	1029044-16-3	0.414	0.294	0.708	Selected for further investigation.
7	dapiprazole	72822-13-0	0.448	0.253	0.702	Selected for further investigation.
8	minaprine	25905-77-5	0.496	0.203	0.700	Selected for further investigation.
9	quinestrol	152-43-2	0.515	0.181	0.696	Selected for further investigation.
10	fenofibric acid	42017-89-0	0.513	0.179	0.692	Selected for further investigation.

Table S4: Summary of the 29 compounds identified by virtual screening.								
**Name**	**CAS identifier**	**Drug class**	**MW / Da *(anhydrous)***	**Hydrogen bonding**		**ClogP**	**Lipinski violations**	**Notes**
				**Donors**	**Acceptors**			
abiraterone acetate	154229-18-2	steroid	391.6	0	3	***5.2***	***1***	
canagliflozin	842133-18-0	flozin	444.5	4	7	3.2	–	
daidzin	552-66-9	isoflavone glucoside	416.4	5	9	0.7	–	
dapagliflozin	461432-26-8	flozin	408.9	4	6	2.3	–	
dapiprazole	72822-13-0	alpha-1 blocker	325.5	0	4	2.4	–	
doxazosin	74191-85-8	alpha-1 blocker	451.5	1	9	2.5	–	
empagliflozin	864070-44-0	flozin	450.9	4	7	2.0	–	
ertugliflozin	1210344-57-2	flozin	436.9	4	7	1.7	–	
fenbufen	36330-85-5	NSAID	236.3	1	3	3.2	–	
fenofibric acid	42017-89-0	fibrate	318.7	1	4	3.9	–	active form
fluralaner	864731-61-3	insecticide	***556.3***	2	10	***5.6***	***2***	
ipragliflozin	951382-34-6	flozin	404.5	4	7	2.5	–	
levosimendan	141505-33-1	calcium sensitizer	280.3	2	6	2.3	–	
licogliflozin	1291094-73-9	flozin	416.5	4	7	1.9	–	
liquiritin	551-15-5	flavanone glucoside	418.4	5	9	0.4	–	
minaprine	25905-77-5	MAOI	298.4	1	5	2.0	–	
pexidartinib	1029044-16-3	kinase inhibitor	417.8	2	7	4.5	–	
phthalylsulfathiazole (PSTZ)	85-73-4	sulfonamide	403.4	3	8	1.1	–	
piribedil	3605-01-4	dopamine agonist	298.3	0	6	1.8	–	
polydatin (piceid)	65914-17-2	stilbenoid glucoside	390.4	***6***	8	1.7	***1***	
quinestrol	152-43-2	steroid	364.5	1	2	***5.3***	***1***	
rafoxanide	22662-39-1	salicylanilide	***626.0***	2	3	***7.0***	***2***	
ramatroban	116649-85-5	TP antagonist	416.5	2	6	2.9	–	
remogliflozin A	329045-45-6	flozin	450.5	4	8	2.2	–	active form
remogliflozin etabonate	442201-24-3	flozin	***522.6***	3	10	3.0	***1***	prodrug
sotagliflozin	1018899-04-1	flozin	424.9	3	6	3.2	–	
tivozanib	475108-18-0	kinase inhibitor	454.9	2	7	4.0	–	
tofogliflozin	1201913-82-7	flozin	386.4	4	6	1.5	–	
velagliflozin	946525-65-1	flozin	395.4	4	6	2.0	–	

**Table S5: Summary of small molecule solubility screen (Methods).**					
**Name**	**ClogP**	**Solubility determination**		**Taylor dispersion analysis (TDA)**	**Decision**
		**Absorbance spectroscopy**	**Capflex**		
YX-I-1	3.1	no light scattering	insufficient signal	insufficient signal	pass
abiraterone acetate	***5.2***	***light scattering***	***spikes (large particles)***	not attempted	***fail***
canagliflozin	3.2	no light scattering	no spikes	R_h = 0.63 nm	pass
daidzin	0.7	no light scattering	no spikes	insufficient signal	pass
dapagliflozin	2.3	no light scattering	no spikes	R_h = 0.60 nm	pass
dapiprazole	2.4	no light scattering	no spikes	R_h = 0.56 nm	pass
doxazosin	2.5	no light scattering	no spikes	R_h = 0.63 nm	pass
empagliflozin	2.0	no light scattering	no spikes	R_h = 0.60 nm	pass
ertugliflozin	1.7	no light scattering	no spikes	R_h = 0.61 nm	pass
fenbufen	3.2	no light scattering	no spikes	unstable baseline	pass
fenofibric acid	3.9	no light scattering	insufficient signal	insufficient signal	pass
fluralaner	***5.6***	***light scattering***	***spikes (large particles)***	not attempted	***fail***
ipragliflozin	2.5	no light scattering	no spikes	R_h = 0.57 nm	pass
levosimendan	2.3	no light scattering	insufficient signal	insufficient signal	pass
licogliflozin	1.9	no light scattering	no spikes	R_h = 0.59 nm	pass
liquiritin	0.4	no light scattering	no spikes	R_h = 0.66 nm	pass
minaprine	2.0	no light scattering	no spikes	R_h = 0.54 nm	pass
pexidartinib	4.5	no light scattering	insufficient signal	insufficient signal	pass
phthalylsulfathiazole (PSTZ)	1.1	no light scattering	insufficient signal	insufficient signal	pass
piribedil	1.8	no light scattering	no spikes	R_h = 0.47 nm	pass
polydatin (piceid)	1.7	no light scattering	no spikes	R_h = 0.63 nm	pass
quinestrol	***5.3***	very weak light scattering	***spikes (large particles)***	not attempted	***fail***
rafoxanide	***7.0***	***light scattering***	insufficient signal	not attempted	***fail***
ramatroban	2.9	no light scattering	no spikes	R_h = 0.55 nm	pass
remogliflozin A	2.2	no light scattering	no spikes	R_h = 0.61 nm	pass
remogliflozin etabonate	3.0	no light scattering	no spikes	R_h = 0.66 nm	pass
sotagliflozin	3.2	no light scattering	no spikes	R_h = 0.68 nm	pass
tivozanib	4.0	very weak light scattering	***spikes (large particles)***	not attempted	***fail***
tofogliflozin	1.5	no light scattering	no spikes	R_h = 0.58 nm	pass
velagliflozin	2.0	no light scattering	insufficient signal	insufficient signal	pass
paracetamol	0.5	no light scattering	insufficient signal	insufficient signal	pass
EGCG	1.2	no light scattering	insufficient signal	insufficient signal	pass

Table S6: Fitted parameters and measures of fit quality from the mechanistic analysis of concentration-dependent IAPP self-assembly kinetics.

**Model name**	**Nucleated polymerization without secondary processes**	**Nucleated polymerization with secondary processes**					
		**Fragmentation**	**Secondary nucleation**		**Fragmentation**	**Secondary nucleation**	
			**Single-step**	**Multi-step**		**Single-step**	**Multi-step**
	θ not a parameter	$\theta = 3/(2n'_2 + 1)$			θ fitted globally		
**Figure panel**	**Fig. S5d**	**Fig. S5e**	**Fig. S5f**	**Fig. S5g**	**Fig. S5h**	**Fig. S5i**	**Fig. S5j**
**Model equations***	eq. [4-7]	eq. [5-9]	eq. [5-8, 10, 11]	eq. [5-8, 10, 12]	eq. [5-9]	eq. [5-8, 10, 11]	eq. [5-8, 10, 12]
n_c	1.56	2.25	1.69	1.69	3.81	2.40	1.89
n_2	–	–	0.83	0.83	–	0.55	3.87
$k_n k_+$ / $\mathrm{M}^{-n_c}.s^{-2}$	29.5	4080	5.71	5.57	2.34×10^{10}	3440	9.76
$k_f k_+$ / $\mathrm{M}^{-1}.s^{-2}$	–	0.0415	–	–	0.298	–	–
$k_2 k_+$ / $\mathrm{M}^{-(n_2+1)}.s^{-2}$	–	–	905	918	–	151	1.65×10^{19} ‡
K_2 / µM	–	–	–	∞ †	–	–	8.85
θ	–	–	–	–	0.324	0.320	0.323
R^2	0.9366	0.9656	0.9692	0.9692	0.9707	0.9727	0.9784
AICc	−5510	−6226	−6357	−6355	−6413	−6497	−6769
ΔAICc	1259	543	512	514	356	272	0 (best fit)

Notes:

* Model equations are listed in **Methods.**

† The value $K_S = \infty$ for multi-step secondary nucleation with $\theta = 3/(2n'_2 + 1)$ represents the fact that the fit repeatedly diverged towards arbitrarily high values of K_S, resulting in a model essentially identical to a single-step nucleation fit. This does not support a single-step nucleation mechanism, as it is simply a result of the constraint on θ making it impossible to simultaneously produce a saturated concentration-dependence and the correct curve shape. Allowing global fitting of θ, to account for late-growth-phase processes, fixed the issue, and allowed a proper comparison between models with a constantly low (single-step) versus satuating (multi-step) concentration-dependence, with the latter strongly favored.

‡ The high apparent value of $k_2 k_+ = 1.65 \times 10^{19}$ in the favored model (multi-step secondary nucleation, globally fitted θ) must be viewed in the context of the high order of the secondary nucleation process ($k_2 k_+$ has dimensions of $\mathrm{M}^{-4.87}.s^{-2}$), ie. the approximate number of monomers that must be assembled together to form a critical nucleus. Multiplying the fitted rate parameter by the IAPP concentration to the appropriate power gives a low actual rate, eg. $\kappa = \sqrt{k_2 k_+ m(0)^{n_2+1}} = 0.00266\ \mathrm{s}^{-1}$ (at 10 µM IAPP) is the rate of autocatalytic amplification of fibril mass due to the positive feedback loop created by secondary nucleation and elongation.

Table S7: Summary of small molecule activity screening (with YX-I-1 and analogues for comparison)									
**Name**	**Dataset**	**Biological repeats**	**Total replicates**	**Fold change in half-time**			***p* value vs. DMSO**		**Notes**
				**Mean**	**Std. dev.**	**SEM**	***t* test**	***U* test**	
DMSO only	preliminary SAR	5	25	(1.000)	0.091	0.018	–	–	
YX-I-1	preliminary SAR	5	25	1.492	0.182	0.036	<0.0001	<0.0001	Known active compound
64882-50-4	preliminary SAR	4	20	0.915	0.093	0.021	0.0035	0.0064	Analogue of YX-I-1 (**Fig. S1**)
ASN00851031	preliminary SAR	4	20	0.897	0.123	0.028	0.0023	0.0044	Analogue of YX-I-1 (**Fig. S1**)
DMSO only	activity screen	9	33	(1.000)	0.088	0.015	–	–	
canagliflozin	activity screen	5	15	1.549	0.223	0.058	<0.0001	<0.0001	Hit (FC > 1.25, $p < 0.0001$)
daidzin	activity screen	3	9	1.176	0.160	0.053	<0.0001	0.0035	
dapagliflozin	activity screen	4	12	1.052	0.069	0.020	0.0734	0.0983	
dapiprazole	activity screen	3	9	1.101	0.075	0.025	0.0031	0.0035	
doxazosin	activity screen	5	15	1.614	0.291	0.075	<0.0001	<0.0001	Hit (FC > 1.25, $p < 0.0001$)
empagliflozin	activity screen	4	12	1.008	0.124	0.036	0.8098	0.8893	
ertugliflozin	activity screen	4	12	1.011	0.127	0.037	0.7513	0.9495	
fenbufen	activity screen	3	9	1.105	0.158	0.053	0.0119	0.0564	
fenofibric acid	activity screen	3	9	0.906	0.121	0.040	0.0121	0.0450	
ipragliflozin	activity screen	4	12	1.229	0.207	0.060	<0.0001	0.0001	
levosimendan	activity screen	2	6	1.028	0.052	0.021	0.4556	0.3907	See **Fig. S6c-e**
licogliflozin	activity screen	4	12	1.067	0.128	0.037	0.0517	0.0880	
liquiritin	activity screen	3	9	1.024	0.151	0.050	0.5481	0.3805	
minaprine	activity screen	3	9	1.019	0.120	0.040	0.6077	0.4321	
pexidartinib	activity screen	3	9	1.187	0.188	0.063	0.0001	0.0054	
phthalylsulfathiazole	activity screen	3	9	1.125	0.174	0.058	0.0046	0.0751	
piribedil	activity screen	3	9	0.976	0.122	0.041	0.5093	0.7404	
polydatin (piceid)	activity screen	3	9	1.119	0.078	0.026	0.0007	0.0015	
ramatroban	activity screen	3	9	1.001	0.024	0.008	0.9877	0.8803	
remogliflozin A	activity screen	4	12	1.083	0.113	0.033	0.0135	0.0352	
remogliflozin etabonate	activity screen	4	12	1.000	0.156	0.045	0.9916	0.5178	
sotagliflozin	activity screen	4	12	1.147	0.260	0.075	0.0064	0.2183	
tofogliflozin	activity screen	4	12	1.083	0.144	0.042	0.0239	0.0930	
velagliflozin	activity screen	2	6	1.057	0.026	0.011	0.1294	0.0833	
paracetamol	activity screen	2	6	1.110	0.073	0.030	0.0065	0.0093	Negative control
EGCG	activity screen	2	6	Strong inhibition, could not determine half-time reliably					Positive control (**Fig. S6b**)

**Table S8: Comparison of fits for mechanistic analysis of the effects of inhibitors on IAPP self-assembly kinetics.**										
**Model**	**Nucleated polymerization with secondary processes (eq. [5-8, 10, 12] in Methods)**									**Favored scenario**
**Process affected**	**Primary nucleation**			**Secondary nucleation**			**Elongation**			
**Fit quality by dataset**	AICc	ΔAICc	Akaike weight	AICc	ΔAICc	Akaike weight	AICc	ΔAICc	Akaike weight	
control + varying YX-I-1	−7591	0	$>99.9\%$	−6581	1010	~0%	−6846	745	~0%	primary nucleation affected
control + varying cana	−7210	0	$>99.9\%$	−6454	756	~0%	−6626	584	~0%	primary nucleation affected
control + varying doxa	−7995	0	$>99.9\%$	−7579	416	~0%	−7727	268	~0%	primary nucleation affected

Table S9a. Cryo-EM data collection statistics for the four samples used for full structure analysis.				
	IAPP control	IAPP + doxa	IAPP + YX-I-1	IAPP + cana
	EMPIAR-12378	EMPIAR-12379	EMPIAR-12380	EMPIAR-12381
**Dataset ID**	Control	Doxa	YX-I-1	Cana
**Small molecule**	none	doxazosin	YX-I-1	canagliflozin
**Magnification**	130,000	130,000	130,000	130,000
**Voltage (kV)**	300	300	300	300
**Detector**	Falcon4-selectris			
**Pixel size (Å)**	0.74	0.74	0.95	0.74
**Total electron dose (e^-/Å^2)**	43	43	50	43
**Exposure rate (e^-/px/s)**	7.6	7.6	10.7	7.6
**Movie frames**	40	40	44	40
**Dose per frame (e^-/Å^2)**	1.07	1.07	1.15	1.07
**Defocus range (mm)**	-1.2 to -2.4	-1.2 to -2.4	-1.4 to -2.6	-1.2 to -2.4
**Movies collected**	2,927	2,959	2,692	2,645
**Initial fibril segments**	749,724	319,880	480,558	132,406

Table S9b. Cryo-EM data collection statistics for the three repeats of the compound-free control.			
	IAPP control	IAPP control	IAPP control
	repeat #1	repeat #2	repeat #3
	EMPIAR-12383	EMPIAR-12384	EMPIAR-12382
**Dataset ID**	ControlRep1	ControlRep2	ControlRep3
**Small molecule**	none	none	none
**Magnification**	130,000	130,000	130,000
**Voltage (kV)**	300	300	300
**Detector**	Falcon4	Falcon4	Falcon4
**Pixel size (Å)**	0.83	0.83	0.83
**Total electron dose (e^-/Å^2)**	46	46	46
**Exposure rate (e^-/px/s)**	7.1	7.1	7.1
**Movie frames**	40	40	40
**Dose per frame (e^-/Å^2)**	1.16	1.16	1.16
**Defocus range (mm)**	-1.5 to -2.7	-1.5 to -2.7	-1.5 to -2.7
**Movies collected**	2,148	2,340	1,536
**Initial fibril segments**	472,565	1,672,881	796,197

Table S9b. Cryo-EM data collection statistics for the repeat reactions with each small molecule.			
	IAPP + doxa	IAPP + YX-I-1	IAPP + cana
	repeat #1	repeat #1	repeat #1
**Dataset ID**	DoxaRep1	YX-I-1Rep1	CanaRep1
**Small molecule**	doxazosin	YX-I-1	canagliflozin
**Magnification**	130,000	130,000	130,000
**Voltage (kV)**	300	300	300
**Detector**	Falcon4-selectris	Falcon4-selectris	Falcon4-selectris
**Pixel size (Å)**	0.95	0.95	0.95
**Total electron dose (e^-/Å^2)**	50	50	50
**Exposure rate (e^-/px/s)**	10.7	10.7	10.7
**Movie frames**	44	44	44
**Dose per frame (e^-/Å^2)**	1.15	1.15	1.15
**Defocus range (mm)**	-1.4 to -2.6	-1.4 to -2.6	-1.4 to -2.6
**Movies collected**	1,947	1,832	2,476

Table S10: Cryo-EM refinement statistics for the four deposited wild-type IAPP structures.

	IAPP-LL	IAPP-LLU	IAPP-LLUU	IAPP-SS (2PF^{S})
	**EMD-51730**	**EMD-51733**	**EMD-51734**	**EMD-51726**
	**PDB-9GZP**	**PDB-9GZS**	**PDB-9GZT**	**PDB-9GZ6**
**EM dataset ID**	**Control**	**Control**	**Control**	**Control**
	Map Refinement			
**Final particle images (#)**	19,396	12,541	12,136	4,015
**Symmetry imposed**	C1	C1	C1	C1
**Map resolution (Å), FSC=0.143**	2.9	3.1	2.9	3.3
**Map resolution range (Å)**	2.9-4.7	3.1-6.8	2.9-4.5	3.3-4.6
**Helical parameters:**				
**Helical twist (°)**	179.88	358.93	179.46	178.24
**Helical rise (Å)**	2.43	4.86	2.43	2.41
**Crossover distance (nm)**	71	80	80	25
	Model Refinement			
**Initial model used (PDB code)**	*de novo*	9GZP	9GZS	6ZRF
**Map sharpening *B* factor (Å^{2})**	-44	-43	-42	-49
**Model resolution (Å), FSC=0.5**	3.0	3.1	3.0	3.6
**Model to map correlation**	0.81	0.84	0.86	0.80
**Model composition:**				
**Non-hydrogen atoms**	2064	3000	3924	2316
**Protein residues**	288	414	540	324
**Solvent molecules**	0	0	0	0
**Layers modelled**	6	6	6	6
**Chains per layer**	2	3	4	2
**IAPP sequence built (chain ID)**	F15-Y37 (A,B)	A13-Y37 (A)<br>N14-Y37 (B)<br>N14-N31 (C)	A13-Y37 (A,B)<br>N14-V32 (C,D)	L12-Y37 (A,B)
***B*-factors (Å^{2}):**				
**Protein**	80	74	28	75
**RMS deviations:**				
**Bond lengths (Å)**	0.007	0.006	0.005	0.003
**Bond angles (°)**	0.922	0.828	0.839	0.952
**Validation:**				
**MolProbity score**	1.5	2.0	1.4	1.3
**Clashscore**	8.8	8.2	6.6	6.3
**Poor rotamers (%)**	0.0	5.2	0.0	0.0
**Ramachandran plot:**				
**Favored (%)**	100.0	100.0	100.0	100.0
**Allowed (%)**	0.0	0.0	0.0	0.0
**Disallowed (%)**	0.0	0.0	0.0	0.0

Table S11: Cryo-EM refinement statistics for the three deposited structures of IAPP fibrils grown in the presence of doxazosin.

	**$IAPP^{+D}$-LL**	**$IAPP^{+D}$-LLU**	**$IAPP^{+D}$-LLUU**
	**EMD-51735**	**EMD-51736**	**EMD-51737**
	**PDB-9GZW**	**PDB-9GZX**	**PDB-9GZY**
**EM dataset ID**	**Doxa**	**Doxa**	**Doxa**
Map Refinement			
**Final particle images (#)**	17,239	16,462	10,492
**Symmetry imposed**	C1	C1	C1
**Map resolution (Å), FSC=0.143**	2.9	3.1	3.0
**Map resolution range (Å)**	2.9-4.9	3.0-5.0	3.0-4.9
**Helical parameters:**			
**Helical twist (°)**	179.39	358.92	179.48
**Helical rise (Å)**	2.43	4.86	2.43
**Crossover distance (nm)**	71	80	83
Model Refinement			
**Initial model used (PDB code)**	9GZP	9GZS	9GZT
**Map sharpening *B* factor (Å^2)**	-54	-44	-30
**Model resolution (Å), FSC=0.5**	2.8	3.0	3.1
**Model to map correlation**	0.86	0.86	0.81
**Model composition:**			
**Non-hydrogen atoms**	2160	3006	3276
**Protein residues**	300	414	456
**Solvent molecules**	0	0	0
**Layers modelled**	6	6	6
**Chains per layer**	2	3	4
**IAPP sequence built (chain ID)**	N14-Y37 (A,B)	Q10-Y37 (A)<br>N14-Y37 (B)<br>L16-T30 (C)	F15-Y37 (A,B)<br>L16-S29 (C,D)
***B*-factors (Å^2):**			
**Protein**	58	78	31
**RMS deviations:**			
**Bond lengths (Å)**	0.002	0.008	0.005
**Bond angles (°)**	0.846	1.037	0.799
**Validation:**			
**MolProbity score**	1.4	1.9	1.4
**Clashscore**	1.7	9.5	7.3
**Poor rotamers (%)**	4.8	3.5	0.0
**Ramachandran plot:**			
**Favored (%)**	100.0	100.0	100.0
**Allowed (%)**	0.0	0.0	0.0
**Disallowed (%)**	0.0	0.0	0.0

References

(1) Taylor, G. I. Dispersion of Soluble Matter in Solvent Flowing Slowly through a Tube. *Proc R Soc Lond A Math Phys Sci* 1953, *219* (1137), 186–203. https://doi.org/10.1098/rspa.1953.0139.

(2) Stender, E. G. P.; Ray, S.; Norrild, R. K.; Larsen, J. A.; Petersen, D.; Farzadfard, A.; Galvagnion, C.; Jensen, H.; Buell, A. K. Capillary Flow Experiments for Thermodynamic and Kinetic Characterization of Protein Liquid-Liquid Phase Separation. *Nat Commun* 2021, *12* (1), 7289. https://doi.org/10.1038/s41467-021-27433-y.

(3) Xu, Y.; Maya-Martinez, R.; Guthertz, N.; Heath, G. R.; Manfield, I. W.; Breeze, A. L.; Sobott, F.; Foster, R.; Radford, S. E. Tuning the Rate of Aggregation of HIAPP into Amyloid Using Small-Molecule Modulators of Assembly. *Nat Commun* 2022, *13* (1), 1040. https://doi.org/10.1038/s41467-022-28660-7.

(4) Dear, A. J.; Meisl, G.; Michaels, T. C. T.; Zimmermann, M. R.; Linse, S.; Knowles, T. P. J. The Catalytic Nature of Protein Aggregation. *J Chem Phys* 2020, *152* (4), 045101. https://doi.org/10.1063/1.5133635.

(5) Michaels, T. C. T.; Dear, A. J.; Knowles, T. P. J. Universality of Filamentous Aggregation Phenomena. *Phys Rev E* 2019, *99* (6), 062415. https://doi.org/10.1103/PhysRevE.99.062415.

(6) Goddard, T. D.; Huang, C. C.; Meng, E. C.; Pettersen, E. F.; Couch, G. S.; Morris, J. H.; Ferrin, T. E. UCSF ChimeraX: Meeting Modern Challenges in Visualization and Analysis. *Prot Sci* 2018, *27* (1), 14–25. https://doi.org/10.1002/pro.3235.

(7) Wilkinson, M.; Xu, Y.; Thacker, D.; Taylor, A. I. P.; Fisher, D. G.; Gallardo, R. U.; Radford, S. E.; Ranson, N. A. Structural Evolution of Fibril Polymorphs during Amyloid Assembly. *Cell* 2023, *186* (26), 5798-5811.e26. https://doi.org/10.1016/j.cell.2023.11.025.
